# Hypervirulent Capsular Serotypes K1 and K2 *Klebsiella pneumoniae* Strains Demonstrate Resistance to Serum Bactericidal Activity and *Galleria mellonella* Lethality

**DOI:** 10.3390/ijms25031944

**Published:** 2024-02-05

**Authors:** Basaier AL-Busaidi, Muna AL-Muzahmi, Zahra AL-Shabibi, Meher Rizvi, Azza AL-Rashdi, Amina AL-Jardani, Robeena Farzand, Zaaima AL-Jabri

**Affiliations:** 1 Microbiology and Immunology Diagnostic Laboratory, Department of Microbiology and Immunology, Sultan Qaboos University Hospital, Muscat 123, Oman; basialbusaidi@gmail.com; 2 Medical Laboratory, Diwan Health Centre, Muscat 130, Oman; m.muzahmi@gmail.com; 3 Department of Genetics, College of Medicine and Health Sciences, Sultan Qaboos University Hospital, Muscat 123, Oman; zahra-alshabibi@hotmail.com; 4 Department of Microbiology and Immunology, College of Medicine and Health Sciences, Sultan Qaboos University, Muscat 123, Oman; 5 Central Public Health Laboratory, Department of Medical Microbiology, Ministry of Health, Muscat 100, Oman; a.rashdi24@gmail.com (A.A.-R.); aksaljardani@gmail.com (A.A.-J.); 6 Department of Genetics and Genome Biology, University of Leicester, Leicester LE1 7RH, UK; rf130@leicester.ac.uk

**Keywords:** hypermucoviscous, *Klebsiella pneumoniae*, hypervirulent, mobile genetic elements, capsule serotypes, virulence, whole genome sequencing, sequence types

## Abstract

Hypervirulent *Klebsiella pneumoniae* (hvKp) is a variant that has been increasingly linked to severe, life-threatening infections including pyogenic liver abscess and bloodstream infections. HvKps belonging to the capsular serotypes K1 and K2 have been reported worldwide, however, very scarce studies are available on their genomics and virulence. In the current study, we report four hypermucoviscous extended-spectrum β-lactamase-producing hvKp clinical strains of capsular serotype K1 and K2 isolated from pus and urine of critically ill patients in tertiary care hospitals in Oman. These strains belong to diverse sequence types (STs), namely ST-23(K1), ST-231(K2), ST-881(K2), and ST-14(K2). To study their virulence, a *Galleria mellonella* model and resistance to human serum killing were used. The *G. mellonella* model revealed that the K1/ST-23 isolate was the most virulent, as 50% of the larvae died in the first day, followed by isolate K2/ST-231 and K2/ST-14, for which 75% and 50% of the larvae died in the second day, respectively. Resistance to human serum killing showed there was complete inhibition of bacterial growth of all four isolates by the end of the first hour and up to the third hour. Whole genome sequencing (WGS) revealed that hvKp strains display a unique genetic arrangement of *k-loci*. Whole-genome single-nucleotide polymorphism-based phylogenetic analysis revealed that these hvKp isolates were phylogenetically distinct, belonging to diverse clades, and belonged to different STs in comparison to global isolates. For ST-23(K1), ST-231(K2), ST-881(K2), and ST-14(K2), there was a gradual decrease in the number of colonies up to the second to third hour, which indicates neutralization of bacterial cells by the serum components. However, this was followed by a sudden increase of bacterial growth, indicating possible resistance of bacteria against human serum bactericidal activity. This is the first report from Oman detailing the WGS of hvKp clinical isolates and assessing their resistance and virulence genomics, which reinforce our understanding of their epidemiology and dissemination in clinical settings.

## 1. Introduction

*Klebsiella pneumoniae* is considered to be the most common cause of hospital-acquired infections (HAIs), accounting for 10% of all nosocomial infections worldwide [1]. Immunocompromised patients are at higher risk of HAIs, comprising 8% to 12% of hospitalized patients, particularly ventilator-associated pneumonia [2]. This can result in life-threatening illnesses with mortality rates ranging from 50% to 100% [3,4]. *K. pneumoniae* have evolved over the years from the classical type into a hypervirulent *K. pneumoniae* (hvKp), which has been observed in many countries around the globe [4]. However, hvKps can also be acquired from the community and have been shown to infect otherwise healthy as well as immunocompetent individuals [5,6]. Emergence of new clonal lineages of hypervirulent strains with co-occurrence of multidrug resistance genotype has been increasing, which is known as multidrug-resistant hvKp (MDR-hvKp). The most common circulating hvKp serotypes include K1, K2, K20, K54, and K57, with K1 and K2 being the most virulent and accounting for 70% of hvKp isolates [7]. HvKp strains produce hypermucoviscous exopolysaccharide coating capsules, which contribute significantly to the pathogenicity and virulence of the bacteria and have been associated with dissemination of severe infections such as pyogenic liver abscess mainly in K1 and ST-23 serotypes [4,5]. Moreover, hvKps can cause other invasive infections, particularly serotypes K2 and ST-65 [4,8]. However, it is well established that the hypermucoviscosity and hypervirulence are two distinct phenotypes and do not necessarily co-exist in virulent strains [9,10].

In addition to the capsule, other virulence factors in *K. pneumoniae* can increase the virulence, including the mucoid phenotype regulators (*rmpA* and *rmpA2*), siderophores including Yersiniabactin *irp2*, *ybt,* and Aerobactin *iucA*, outer membrane porin (KpnO), and phospholipase D family protein (PLD1) [11]. Furthermore, there are other virulence factors which have not been thoroughly characterized, including efflux pumps, iron transport systems, and genes that are involved in allantoin metabolism [12,13,14]. In addition, some studies have demonstrated that the virulome of the same species could change depending on the host factors [15,16]. Some factors that expose hospitalized individuals to colonization and infection include admission into the intensive care unit (ICU), poor adherence to infection control strategies, prolonged use of invasive devices, and broad-spectrum antibiotics. Moreover, host-related factors such as immunocompromised state, especially diabetics and alcoholics, could increase the risk of colonization of virulent *K. pneumoniae* [17].

Apart from the intrinsic resistance to ampicillin in hvKps, the antibiotic resistance phenotype in hvKps is still not very prevalent, and the reason is still unclear [4]. However, there has been an increasing trend in resistance in hvKp isolates due to dissemination via mobile genetic elements such as types of New Delhi metallo-β-lactamases (NDMs), oxacillinases β-lactamases (OXA-48), and *K. pneumoniae* carbapenamases (KPCs) [13,16,17]. Recently, the hvKp serotypes K1 and K2 have been detected from clinical isolates in Oman [8]. Therefore, this study aimed to characterize their virulence and antibiotic resistance genomically and to further study host responses against infection with *K. pneumoniae* K1 and K2 serotypes circulating in Oman.

## 2. Results

### 2.1. Sequence Types of K. pneumoniae Isolates

**Figure 1:**
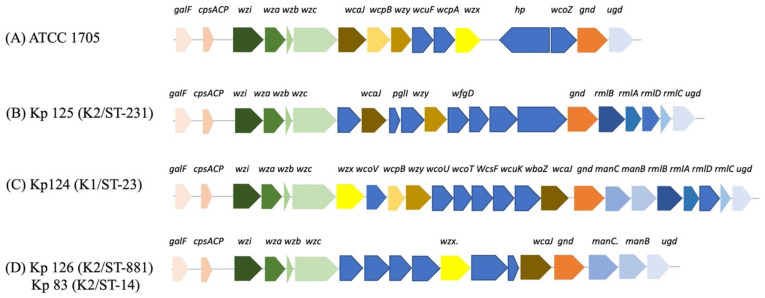
*k-loci* gene arrangement of hvKP isolates. (**A**) ATCC 1705, (**B**) Kp 125 (K2/ST-231), (**C**) Kp 124 (K1/ST-23, (**D**) Kp 126 (K2/ST-881), and Kp 83 (K2/ST-14). Gene names are indicated above the colored arrows, and each group of genes with a shared function or operon are colored with the same colors. Chromosomal genes are conserved in all isolates including capsular synthesis, capsule translocation, and surface assembly genes *galF, ORF2, wzi, wza, wzb, wzc,* and *gnd. wcoZ* is uniquely found in the *K. pneumoniae* ATCC 1705 control strain and one additional isolate from our study (Kp102) (Supplementary Figure S5). GDP-D-mannose synthesis genes *manC* and *manB* (**C**,**D**), and glucose conversion genes *rmlA, rmlB, rmlC, and rmlD* (**B**,**C**).

Initial PCR screening revealed that only 4 out of 129 (3%) *K. pneumoniae* isolates were hypervirulent capsular strains, namely Kp124 (K1/ST-23), Kp 125 (K2/ST-231), Kp 126 (K2/ST-881), and Kp 83 (K2/ST-14) (Figure 1).

Three of these isolates were invasive *K. pneumoniae* strains with hypermucoviscous phenotype (HMV *K. pneumoniae*), and one of these (K2/ST-881) was a carbapenemase-producing (CRE) isolate. WGS analysis was performed on these four isolates in addition to the other isolates for determining the ST and *k-loci* composition. MLST was performed on the isolates from WGS data to determine their sequence type (ST) using the CGE website (Table 1) [18]. One isolate from a pyogenic infection belonged ST-23, similar to global trends associating these features [7]. On the other hand, the three other isolates belonged to three different STs, including ST-14, ST-231, and ST-881 (Table 1). One-third (32%, *n* = 10) of the isolates belonged to ST-231 and 19% of the isolates (*n* = 6) belonged to ST-395 (Table 1). Moreover, 6% of the isolates (*n* = 2) belonged to ST-23 and another 6% of the isolates (*n* = 2) belonged to ST-405.

### 2.2. k-loci Analysis of hvKp Isolates

It was shown that the isolates with unique *k-loci* genetic arrangements belonged to the hvKp isolates with serotypes K1/ST-23, K2/ST-231, K2/ST-881, and K2/ST-14 (Table 2).

The phenotypic antimicrobial susceptibility profiles by disk diffusion of the hvKp isolates are presented in Table 3 and Table 4. All hvKp isolates in this study (Kp 83, Kp 125, and Kp 126) that were K2 positive and Kp 124 that was K1 positive were ESBL producers. Kp 83 (K2/ST-14) showed resistance to AMP, TZP, FEP, CTX, IMP, CAZ, and CIP. Kp 126 (K2/ST-881) was resistant to AMP, FEP, CTX, IMP, CAZ, and CIP. Meanwhile, Kp124 (K1/ST-23) was resistant to AMP, TZP, FEP, CTX, FOX, and CAZ. On the other hand, Kp 125 (K2/ST-231) was a KPC producer with resistance to AMP, TZP, FEP, CTX, and CAZ.

### 2.3. Plasmid Compositions of hvKp K1/ST-23 (Kp 124), K2/ST-231 (Kp 125), K2/ST-881 (Kp 126), and K2/ST-14 (Kp 83)

Whole-genome sequences of K1/ST-23, K2/ST-231, K2/ST-881, and K2/ST-14 isolates comprised 5,569,468 (Kp 125 K2/ST-231) bases, 5,572,070 bases (K1/ST-23), 5,638,661 bases (Kp 126 K2/ST-881), and 5,447,756 bases (Kp 83 K2/ST-14) with 57% GC content in all strains (Supplementary Table S7). The isolate Kp 125 belonging to K1/ST-231 contained seven plasmids: pColKP3, which carried one copy of *OXA-_181_*, and *KPC-3*, conferring carbapenem and cephalosporin resistance by mechanism of antibiotic inactivation. The other plasmids were pIncFII(pAMA11 67-NDM-5), carrying New Delhi metallo-β-lactamase (NDM)-producing enzymes, and pIncFIB(pQil). Isolate belonging to K2/ST-23 carried three plasmids, including pIncFIB(pQil), containing KPC-3, which has been attributed to the global spread of *bla*-_KPC_.

In K1/ST-23, Col440II shared 100% identity with the reference plasmid (CP023921). On the other hand, pIncFIB(K) shared 98% identity with the reference plasmids. Isolate belonging to K2/ST-231 had three plasmids, which were IncFIB(pQil), IncFII(pAMA1167-NDM-5), and RepB. pRepB is a megaplasmid (244,152 bp) that is associated with the IncFIB family and has been known to integrate massive accessory modules. In ST-231, pRepB carried a very large composite transposon (15,832 bp), insertion sequences (ISEc27), IS102-like among other mobile elements like integrons, and MDR-conferring genes [19].

Isolate Kp 83 (K2/ST-14) had four plasmids, including pIncFIA (HI1), which carried resistance genes for several antibiotic categories such as tetracyclines, phenicols, folate pathway antagonists, and aminoglycosides. pIncFIB(K) harbored resistance genes against sulfonamides, macrolides, and beta lactams. pIncFIB(pNDM-Mar) carried antimicrobial genes against phenicols, beta lactams, quinolones, and aminoglycosides. Finally, pIncR carried antibiotic genes conferring resistance to beta lactams, quinolones, and aminoglycosides (Table 3).

### 2.4. Virulence-Associated Genes of hvKp Isolates

WGS of Kp 124 (K1/ST-23), Kp 125 (K2/ST-231), Kp 126 (K2/ST-881), and Kp 83 (K2/ST-14) were compared to the reference genomes of *K. pneumoniae* strain RJF293 (accession number CP014008.1), which belongs to serotype K2 assigned to ST-374. WGS analysis revealed that genes encoding for pathogenicity were conserved in the analyzed strains at the same relative chromosomal position. There are four main virulence classes in *K. pneumoniae,* including the capsule, lipopolysaccharide (LPS), siderophores, and fimbriae (Supplementary Table S8). Enterobactin (*entB)* was detected in all the four hvKp isolates, whereas aerobactin (*iucA*) was found in Kp 124 (K1/ST-23), Kp 125 (K2/ST-231), and Kp 126 (K2/ST-881) but not Kp 83 (K2/ST-14). Yersiniabactin (*irp2* and *ybt*) and salmochelin (*iroB*) were missing in all isolates. Capsule biosynthesis gene *rcsB* was detected in all isolates but not the regulators *rmpA* and *rmpA2*. LPS, adhesins, and type 3 fimbriae were detected in all isolates.

### 2.5. Human Serum Bactericidal Killing Susceptibility and G. mellonella Lethality

**Figure 2:**
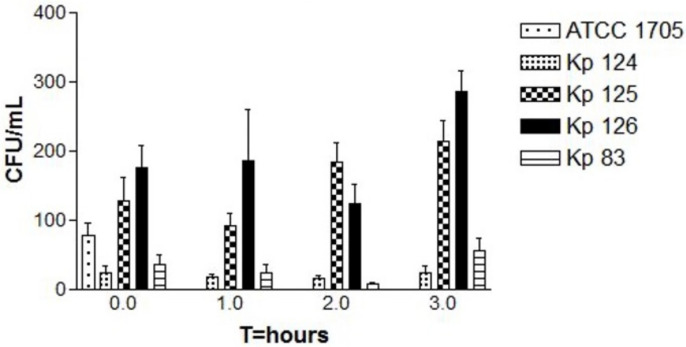
Bactericidal activity of human serum samples against hvKp clinical strains. The CFU counts of all strains including the control were tested at four time points, T = 0, T = 1, T = 2, and T = 3 (time in hours). Each strain represents the mean for three replicates: *K. pneumoniae* ATCC 1705 (control), K1/ST-23(Kp 124), K2/ST-231 (Kp 125), K2/ST-881 (Kp 126), and K2/ST-14 (Kp 83). The *p*-value was 0.0007 for CFU counts, which is statistically significant as shown by one-way ANOVA. Error bars represent SEM. A slight dip in the bacterial growth at T = 2 is followed by a slight to significant increase in the bacterial growth in all three replicates at T = 3 for Kp 125, Kp 126, and Kp 38.

In isolate Kp 124 (K1/ST-23) (Figure 2), there was a slight decrease in the growth, indicating the death of bacterial cells, followed by a slight increase in the bacterial growth at T = 3. In isolate Kp 125 (K2/ST-231), in the first hour, there was a decrease in the number of colonies, however, in the second and third hours, there was a significant increase in bacterial growth. In isolate Kp 126 (K2/ST-881), there was a decrease in growth of bacteria in the first hour, but in the second and third hour there was a noticeable increase in the number of colonies, which was higher compared to the previous two isolates. In Kp 83 (K2/ST-14), there was a slight decrease in the number of colonies in the first and second hours. However, after the second hour, and by the third hour, there was a dramatic increase in the bacterial growth. In all replicates of the four tested isolates, the *p*-value showed statistical significance (*p*-value of 0.0007).

**Figure 3:**
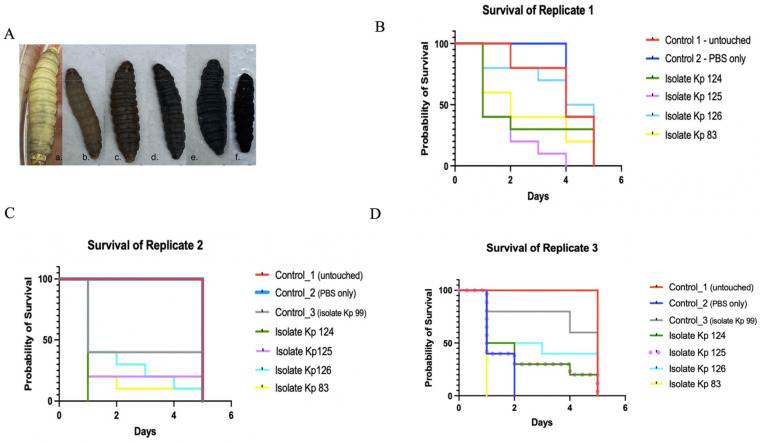
Kaplan–Meier survival curve of *K. pneumoniae* clinical isolates. (**A**) Phenotypic characteristics of *G. mellonella* larvae pre and post injection and throughout the 5 observation days. a is the healthy larvae at day 0 of the experiment, b–f shows gradual death of the larvae with blackening from day 1-5. (**B**–**D**) Survival of *G. mellonella* larvae infected with K1/ST-23(Kp 124), K2/ST-231 (Kp 125), K2/ST-881 (Kp 126), and K2/ST-14 (Kp 83) in comparison to controls 1 and 2 over the course of 5 days. Control 1—untouched, uninfected larvae are represented in red. Control 2—larvae injected with PBS are represented in blue. A third control, Kp 99 in replicate (**C**,**D**), was used, which was a non-hypervirulent *K. pneumoniae* isolate, just to compare the rate of survival to the hypervirulent strains. Isolate Kp 124 was represented in green, isolate Kp 125 in the purple straight line in (**C**) and dotted in panel (**D**) for clarity, isolate Kp 126 in cyan, and isolate Kp 83 in yellow as indicated in the color legend on the right side of each image.

To correlate between resistance to serum killing and virulence, the same strains were injected into *G. mellonella* larvae. The survival curves of the three replicates were shown separately for comparison (Figure 3). Isolates Kp 124 (K1/ST-23) and Kp 83 (K2/ST-14) seemed to be the most virulent isolates, as most larvae died in the first two days. However, isolates Kp 125 (K2/ST-231) and Kp 126 (K2/ST-881) seemed to be less virulent, as the number of larvae gradually decreased throughout the experiment. In the three replicates (Figure 3B), the Control 1 larvae consisted of the untouched larvae. In the three replicates of the control group, there was a gradual decline in the survival of the larvae from day 2 to 5, as the first larva died in the second day in one of the replicas, while in the other two replicates the larvae died at the end of day 5.

In Control 2, larvae that received only PBS, there was also a gradual decline in the survival of the larvae throughout the 5 days in replicate one, however, no death was noticed in the second replicate until the larvae slowed down in motility as cocoon formation commenced. However, in the third replicate, there was 100% death, which could suggest that the batch contained mature larvae in that control group. In Control 3 larvae, consisting of larvae injected with isolate Kp 99 that is negative for all hypervirulent capsular serotypes, on the first day, about 20–50% of the larvae died, with no death in the remaining 50–60% of larvae until the end of the experiment as observed in the three replicates. In the strain Kp 124 (K1/ST-23) test group, more than 50% in two replicates and 100% in the other replicate of the larvae died on the first day of the experiment. The remainder of larvae were dead by the fourth day. For the strain Kp 125 (K2/ST-231) test group, about 50–75% of the larvae died on the second day, and by the fourth day, all larvae were dead in all three replicates. In the isolate Kp 126 (K2/ST-881) test group, the death ranged between 25% and 60% of the larvae on the first day, but overall, the survival rate declined gradually throughout the 5 observation days in the three replicates. In isolate Kp 83 (K2/ST-14), more than 50–100% of larvae died by the second day of the experiment in the three replicates.

### 2.6. Genetic Relatedness to Global Isolates

**Figure 4:**
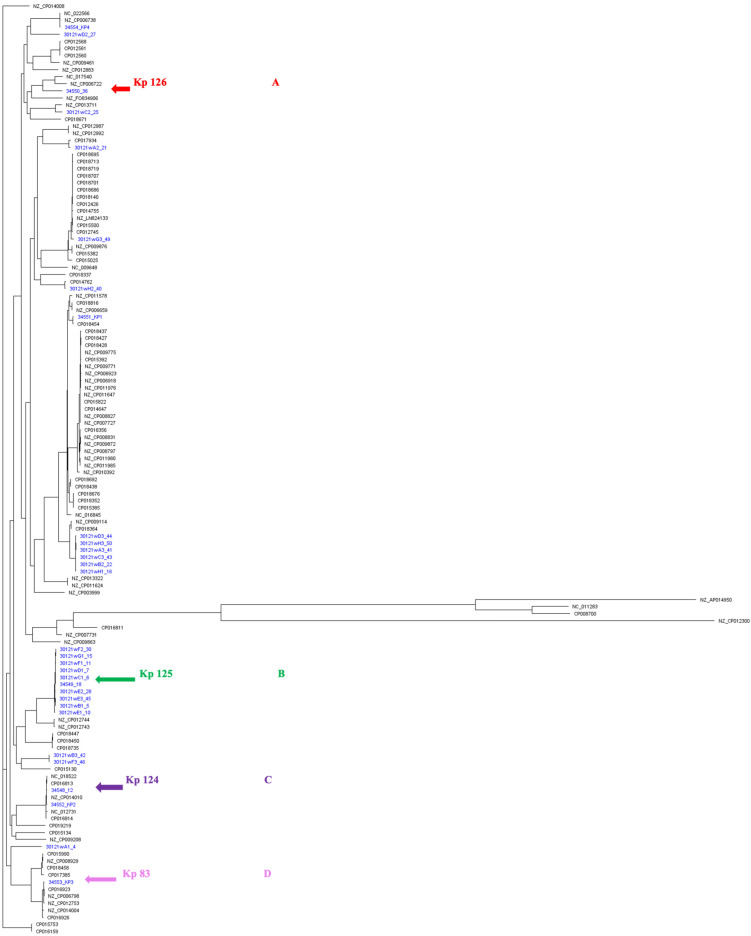
A whole-genome single-nucleotide polymorphisms (SNPs) calling phylogenetic tree of *K. pneumoniae* isolates (**A**) (*n* = 131). A maximum-likelihood tree showing genetic relatedness between *K. pneumoniae* strains based on WGS data. The strains are labeled with serial numbers and grouped according to their STs. *K. pneumoniae* RJF293 (GenBank accession number CP014008) was used as a reference. The isolates starting with NZ and CP (in black) are global published isolates retrieved from Genbank and were used for comparison. The isolates in blue are strains from this study. The hypervirulent isolates are pointed at with arrows: red for K2/ST-881 (Kp126), green for K2/ST-231 (Kp125), purple for K1/ST-23 (Kp 124), and pink for K2/ST-14 (Kp83). iTOL was used to visualize and annotate the tree. Clades are indicated in (**A**–**D**). This figure compares the local strains in this study (represented in blue) as well as global strains, which clearly demonstrates the diverse clonality of clinical isolates from Oman, belonging to various STs.

A whole-genome SNPs calling phylogenetic tree of 131 isolates including 30 strains in this study (from CPHL and Kp 83, represented in blue) as well as 101 strains from Genbank global strains (represented in black) [17] were plotted to compare genetic relatedness (Figure 4).

**Figure 5:**
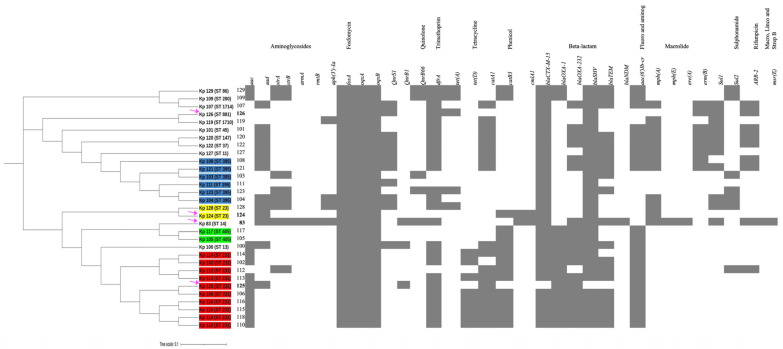
A whole-genome single-nucleotide polymorphisms (SNPs) calling phylogenetic tree of *K. pneumoniae* isolates (*n* = 31) and heat map of resistance-encoding genes distribution and their antimicrobial classes. A maximum-likelihood tree showing genetic relatedness between *K. pneumoniae* strains based on WGS results. The isolates are grouped and highlighted according to their STs (blue = ST 395, red = ST 231, green = ST 405, yellow = ST 23, unhighlighted = miscellaneous STs). The hypervirulent isolates K1/ST-23 (Kp 124, K2/ST-231 (Kp 125), K2/ST-881 (Kp 126), and K2/ST-14 (Kp 83)) are pointed at using magenta arrows. *K. pneumoniae* RJF293 (GenBank accession number CP014008) reference was used. iTOL was used to visualize and annotate the tree. Hypervirulent Kp 83 (K2/ST-14) and Kp 124 (K1/ST-23) are branching from the same clade but belong to two different clusters, C and D, respectively. The relation between their STs and resistance phenotype indicates the diversity in the acquired antimicrobial genes in these hypervirulent strains.

A separate phylogenetic tree of 31 isolates for a close-up inspection, including the hypervirulent isolates of this study Kp125 (K2/ST-231), Kp 126 (K2/ST-881), and Kp 83 (K2/ST-14), as well as 27 other non-hypervirulent *K. pneumoniae* local clinical isolates (Figure 5), was created. The unique STs were highlighted in different colors. Group A in blue is representing isolates with ST-395, group B in yellow belongs to ST-405, group C in green belongs to ST-23, group D in red represents ST-231, and finally the unhighlighted isolates belong to miscellaneous STs. The hypervirulent isolates Kp 124 (K1/ST-23), Kp 125 (K2/ST-231), Kp 126 (K2/ST-881), and Kp 83 (K2/ST-14) are indicated with magenta arrows. Hypervirulent Kp 83 (K2/ST-14) and Kp 124 (K1/ST-23) are branching from the same clade but belong to two different clusters, C and D, respectively. Following construction of the *k-loci* in Kaptive, it was evident from the phylogenetic tree that the clinical strains with similar STs have high similarity in the genetic arrangement of *k-loci* (Figure 1 and Figures S3–S30). The hypervirulent serotypes positive for K2, namely K2/ST-231 (Kp 125), K2/ST-881 (Kp 126), and K2/ST-14 (Kp 83), exhibit diversity in their STs, resulting in distinct *k-loci* gene compositions.

The *K. pneumoniae* capsular synthesis loci, *k-loci,* in all our isolates generally consisted of the following conserved genes, including *galF*, *ORF2 (cpsACP)*, *wzi*, *wza*, *wzb*, *wzc*, and *gnd,* which are chromosomally encoded core and similar to what has been reported in the literature [18,19] (Figure 1), as these are necessary for capsular synthesis along with other genes. Moreover, *galF*, *ORF2,* and *gnd* are involved in carbohydrates metabolism, and *wzi (orfX)*, *wza*, *wzb*, and *wzc* are responsible for the capsule translocation and surface assembly [20,21]. In this study, the ICEfinder showed that Kp 125 (K2/ST-231), Kp 126 (K2/ST-881), and Kp 83 (K2/ST-14) had 7, 3, 2, and 1 putative type 4 secretion systems with putative ICEs, respectively (Supplementary Figure S33).

The hypervirulent strains carried a wide range of acquired antimicrobial genes (Figure 5). Kp 83 (K2/ST-14) carried a variety of resistance-conferring genes as compared to the other isolates, except for *qnrS1*, *blaOXA-2*, *blaAP,* and *tet(A)* genes. Kp 124 (K1/ST-23) carried the least number of resistance-conferring genes and harbored no resistance genes for some antibiotic classes including folate pathway antagonist, quaternary ammonium compounds, streptogramin b, rifamycin, aminocyclitol, macrolide, lincosamide, and tetracycline. Moreover, isolate Kp 125 (K2/ST-231) carried resistance genes for all antibiotic classes in Figure 4, except glycosamide and tetracycline. In addition, Kp 126 (K2/ST-881) seemed to lack resistance-conferring genes to aminoglycosides, amphenicol, streptogramin b, rifamycin, aminocyclitol, macrolide, and lincosamide. 

## 3. Discussion

The hvKp are known to be associated with increased virulence as well as with severe human infections [22]. Only 3% of the clinical isolates tested positive for K1 and K2 during the same period in 2019 at Sultan Qaboos University Hospital (SQUH), from urine and pus samples, and one K2 case was reported in 2017 from a urine specimen. This finding is consistent with several studies, which showed that the most commonly circulating hypervirulent capsular serotypes are K1 and K2, which has been attributed to disruption of epithelial and mucosal barriers such as endotracheal tubes, catheters, and surgical wounds in hospital settings [21,23,24,25,26]. Phenotypically, all the hypervirulent isolates in this study were ESBL producers, which are known to be associated with increased length of stay and mortality rates. In contrast to previous studies, the prevalence rate of ESBL producers was higher in non-hypermucoviscous *K. pneumoniae* isolates [20,21,23,24,25,26]. There is a high possibility of opportunistic infections in our patients included in this study, as the majority are immunocompromised.

The *k-loci* of the capsular operon share similar genetic organization, in which major operons are conserved in most isolates with few exceptions. MLST analysis showed that most of the isolates with similar STs had similar gene arrangements with a similar reference genome in Kaptive. Moreover, these strains were in the same cluster in the whole-genome SNP calling phylogenetic tree. The remaining strains belonged to miscellaneous STs and, therefore, variable *k-loci* genetic arrangement figures were observed. To the best of our knowledge, this observation has not been previously reported, making this study the first in Oman to indicate a significant correlation between genetic composition of the *k-loci* and their STs.

In addition to the conserved genes in the *k-loci*, there are other genes that are commonly present in the operon, including *manC* and *manB,* which are involved in the synthesis of GDP-D-mannose as well as RmlA, RmlB, RmlC, and RmlD proteins involved in the conversion of glucose 1-phosphate to dTDP-L-rhamnose [27]. Moreover, additional virulence determinants such as aerobactin, but not yersiniabactin, salmochelin, or enterobactin, enables the growth and survival of hvKp ex vivo and in vivo [4]. The presence of a wide variety of virulence-conferring genes in the isolates of this study indicates that these hvKp strains were well equipped with the virulence machinery, which could, under the right conditions, enable a hypervirulent phenotype.

Previous studies proved that hypervirulent *K. pneumoniae* strains are associated with increased antibiotic resistance, therefore, recognizing their global dissemination is an urgent priority [5,28,29,30,31]. There has been an increase in reporting MDR in hvKp clinical strains over the last few years, therefore designated as MDR-hvKp. This was attributed to the horizontal gene transfer mediated by plasmids (type I and type II) [5,22,32,33]. For example, type I plasmids were reported to carry *bla*_NDM-1_ and *bla*_KPC-3_ [20,21,23,24]. In silico detection of plasmids using plasmidFinder demonstrated that the plasmids in these strains carry mobilizable pathogenicity and resistance-conferring genes [14,15] (Table 3). While most of these plasmids share common sequences, there are wide areas of mosaic genetic structures that might indicate multiple recombination events (Supplementary Figures S31 and S32).

According to ResFinder and CARD online tools findings, the hypervirulent strains in this study including Kp 124 (K1/ST-23), Kp 125 (K2/ST-231), Kp 126 (K2/ST-881), and Kp 83 (K2/ST-14) were in fact highly resistant, carrying a wide variety of antibiotic resistance genes for multiple antimicrobial classes. All these strains carried incompatibility group (Inc) FIB plasmids that are known to encode both virulence and antimicrobial resistance genes in a wide variety of intestinal bacteria including *E. coli, Salmonella,* and *Klebsiella* spp. including IncFIA and IncFIB that represent one of the most common plasmid types that plays a major role in the dissemination of AMR in *Enterobacteriaceae* [34,35,36,37,38,39]. Furthermore, IncFIB (K) was carried by both Kp 83 (K2/ST-14) and Kp 126 (K2/ST-881), which suggested the likelihood of HGT of these plasmids between different bacteria [40]. Moreover, Kp 125 (K2/ST-231) carried a ColKP3 plasmid that is known to harbor OXA-_232_ carbapenemase responsible for the rapidly increasing carbapenem resistance in *K. pneumoniae* strains worldwide [41]. Moreover, Kp 125 (K2/ST-231) and Kp 124 (K1/ST-23) both carried IncFIB(pQil), which is known to be harbored by *K. pneumoniae* and other pathogens responsible for nosocomial infections [42]. This finding suggests the likelihood of HGT of this plasmid amongst *K. pneumoniae* bacterial isolates since both Kp 125 (K2/ST-231) and Kp 124 (K1/ST-23) were isolated in the same period in 2019 in SQUH.

SNP calling phylogenetic trees performed on WGS data show that *K. pneumoniae* strains isolated from different geographical regions (*n* = 131) were arranged with distinct clustering [40,41,42,43]. The strains in our study were found to be phylogenetically diverse, belonging to various STs. The hvKps Kp 124 (K1/ST-23) and Kp 125 (K2/ST-231) are present in a clade A, whereas Kp126 (K2/ST-881) is in clade B. The close-up tree showed that our strains were grouped in four main clusters, highlighted with different colors, indicating the diverse groups of strains circulating in our local hospitals. However, there was clustering in these four main groups, indicating a possibility of dissemination at a possibly larger scale [43,44]. Isolates belonging to ST-231 have been associated with MDR spread in Europe, with the potential to cause a future epidemic, posing a public threat. These strains produce OXA-_232_ as well as RmtF, which are associated with high-level carbapenem resistance [45,46]. Furthermore, *K. pneumoniae* belonging to ST-395 are associated with the production of OXA-_48_, which is the most common enzyme causing healthcare-associated infections globally. The OXA-_48_ enzyme hydrolyses carbapenems and shows weak activity against extended-spectrum cephalosporins such as cefepime and ceftazidime, making it difficult to treat these infections [47,48]. This clone of *K. pneumoniae* has been detected in many countries of the Middle East, including Oman, Yemen, and Saudi Arabia [49]. Furthermore, Kp 117 and Kp 105 belong to ST-405 (blue cluster) and have also been associated with the production of OXA-48 just like clones belonging to ST 395 and thus have been responsible for many hospital-associated outbreaks and war-related outbreaks (in Germany), and some of which are resistant to colistin [50,51,52].

Several studies have shown that infection of *G. mellonella* with *K. pneumoniae* has resulted in host responses similar to the innate immune responses in murine models including cell death, inhibition of phagocytosis, and antimicrobial peptide production. Furthermore, *G. mellonella* can differentiate between pathogenic and non-pathogenic *Klebsiella* strains and showed that the more virulent strains were associated with increased survival and host cellular damage as compared to avirulent strains [53].

In the present study, it was shown that Kp 124 (K1/ST-23) was the most virulent, as 50% of the larvae died on the first day, followed by K2/ST-231, where 75% of the larvae died on the second day, and Kp 83 (K2/ST-14), where 50% of the larvae died on the second day. Similarly, in Europe, K1-positive *K. pneumoniae* belonging to ST-23 have been associated with severe and fatal infections associated with liver abscesses [3,25].

However, in Kp 126 (K2/ST-881), there was a gradual decline in the number of larvae over the course of 5 days, perhaps making it the least virulent, as the death was slower and steadier. It was expected for Kp 126 (K2/ST-881), although it was a hypervirulent strain, that it belonged to ST-881, which, according to the literature, has not been associated with severe human infections, as compared to the other STs of Kp 83 (K2/ST-14), Kp 124 (K1/ST-23), and Kp 125 (K2/ST-231). The *G. mellonella* experiment was repeated three times to allow for comparison and to check how the quality of larvae might affect the results. The results of the experiments indicated the higher virulence of hypervirulent K1 and K2 capsular serotype isolates as compared to non-hypervirulent strains [1,54]. However, the main limitation in this assay was due to shipping and delivery restrictions caused by the COVID-19 pandemic, as the larvae used in this study were subjected to variations in temperatures and storing conditions.

Human serum resistance experiments were performed for the hypervirulent strains Kp 124 (K1/ST-23), Kp 125 (K2/ST-231), Kp 126 (K2/ST-881), and Kp 83 (K2/ST-14). For each isolate, three replicates were performed for accurate comparison and reproducibility. There was complete inhibition of bacterial growth in all four isolates by the end of the first hour and up to the third hour. This was due to the presence of complement proteins and lysozyme, which have a bactericidal effect [55]. Furthermore, an unexpected pattern of bacterial growth was observed in all replicas for all four strains. For Kp 124 (K1/ST-23), there was a gradual decrease in the number of colonies up to the second hour, which indicates neutralization of bacterial cells by the serum components; however, in the third hour, there was a sudden splurge of bacterial growth. For Kp 125 (K2/ST-231), the same decrease in bacterial growth was noticed by the end of the first hour, but there was a dramatic increase in the bacterial growth starting from the second hour, unlike in Kp 124 (K1/ST-23). Furthermore, for Kp 126 (K2/ST-881) and Kp 83 (K2/ST-14), similar patterns of sudden growth of bacterial cells commenced in the third hour. Therefore, the sudden increase in the bacterial growth despite the unfavorable serum environment for growth indicates possible resistance of bacteria against human serum bactericidal activity [56]. Human serum, unlike blood, contains antibodies and complement proteins that contribute to the neutralization of bacteria but does not contain immune cells such as phagocytes. Previous studies discuss the bactericidal activity that human serum possesses against Gram-negative bacteria including *K. pneumoniae.* Moreover, they highlight that the resistance against human serum may be a significant virulence factor. Therefore, the strains used in this assay might have acquired the resistance against human serum as one of their virulence factors [55]. Furthermore, earlier studies have shown that *K. pneumoniae* LPS O antigen and the capsule polysaccharide contribute strongly to the resistance against human serum antibodies and complement proteins. The isolates tested in this experiment were hypervirulent and possessed a thick capsule, therefore, their resistance to human serum could be explained by this theory [55,56,57,58]. In future studies, it would be worthy to test the hypervirulent strains of this study in human blood that contains immune cells such as phagocytes to investigate how it compares to human serum. In previous studies, *K. pneumoniae* has shown its ability to resist killing by phagocytosis in human blood killing assays [59].

## 4. Materials and Methods

### 4.1. Bacterial Isolates

This study was commenced after obtaining approval by the Medical Ethics Research committee (MREC#1896), College of Medicine and Health Sciences, Sultan Qaboos University, Muscat, Oman. Initially, we screened 129 *K. pneumoniae* clinical isolates for K1, K2, K20, K54, and K57 from various samples (urine, wound, tracheal aspirate, blood, sputum) from patients from Central Public Health Laboratories (CPHL) (Supplementary Figures S1 and S2) representing various areas from Oman collected between 2015 and 2020. Another 30 isolates were screened from Sultan Qaboos University Hospital (SQUH), Muscat, Oman, collected between 2019 and 2021 (Supplementary Table S1). PCR preliminary data are presented in Supplementary Tables S5 and S6, including PCR cycling conditions and primers. Colonies were collected from purity plates of Cystine Lactose Electrolyte-Deficient (CLED) agar (Oxoid, Basingstoke Hampshire, UK). These colonies were used to make frozen stock in beads-containing cryotubes and processed according to the manufacturer’s instructions (Mast Diagnostics, Derby, UK). The samples were frozen at −80 °C for future use.

### 4.2. String Test for Hypermucoviscosity

An inoculation loop was used to stretch a colony on a CLED agar plate (Oxoid, Basingstoke Hampshire, UK), and the immediate formation of a viscous string of >5 mm in length was indicative of a hypermucoviscous strain of *K. pneumoniae* [60].

### 4.3. Antibiotic Susceptibility Testing

Antimicrobial susceptibility profiles of the isolates were carried out using the disk diffusion method [61]. Firstly, from an overnight pure culture, three to five colonies were suspended in 5 mL of normal saline (Fisher Scientific, Loughborough, UK). The suspension was adjusted to 0.5 McFarland (approximately 1–2 × 10^8^ CFU/mL) using a CrystalSpec nephlometer (BD Diagnostics, Baltimore, MD, USA) following the manufacturer’s protocol. The antibiotic disks included ampicillin (AMP 10 mg), piperacillin/tazobactam (TZP 110 mg), cefepime (FEP 30 mg), cefotaxime (CTX 30 mg), cefoxitin (FOX 30 mg), ceftazidime (CAZ 30 mg), imipenem (IPM 10 mg), meropenem (MEM 10 mg), amikacin (AK 30 mg), gentamicin (CN 10 mg), and ciprofloxacin (CIP 5 mg) (Liofilchem, Roseto degli Abruzzi, Italy and BioMérieux, Voie Romaine, Craponne, France). The susceptibility assay was carried out according to the CLSI standards [62], placed on the nutrient agar plates (Oxoid, Hampshire, UK), and inoculated using sterile forceps. The plates were then incubated for 18–24 h at 37 °C. *E. coli* (ATCC 25922) and *P. aeruginosa* (ATCC 27853) were used as susceptible control strains. The readings were taken after 18–24 h, and the interpretive categories and zone diameter breakpoints are listed in Table 4.

### 4.4. PCR Screening

Initial PCR screening of MDR *K. pneumoniae* was performed for the purpose of assessing the prevalence of hvKp capsular serotypes (Supplementary Figure S1). Initial screening of MDR *K. pneumoniae* revealed that four isolates displayed hvKp phenotype. These four isolates were isolated mainly from urine and one from a wound swab, which were collected during possible cross-transmission episodes in SQUH, Muscat, Oman between 2019 and 2020. We conducted a comparison of the *k-loci* in these isolates to known *K. pneumoniae* references in the database using the Kaptive 2.0 online tool to analyze similarities and variabilities [9]. The genetic arrangement of different *k-loci* in each isolate as well as percentage of similarity between the isolate to known references showed that most of the screened *K. pneumoniae* clinical isolates have similar genetic arrangement (Supplementary Figures S3–S30). The matches of “Good” or “very good” confidence were reported based on the 90% to 95% identity match.

### 4.5. DNA Extraction and WGS

DNA was extracted from an overnight culture using the QIAamp DNA Mini Kit (Qiagen, Germany) for whole-genome sequencing. The manufacturer’s protocol was followed with slight modifications. For highly mucoid *K. pneumoniae* isolates, a pre-lysis step was performed, which includes the preparation of a pre-lysis buffer consisting of 100 μL of Tris//EDTA (TE) buffer (ThermoFisher Scientific, Waltham, MA, USA), 1.00 μL lysozyme 10 mg/mL (final concentration 0.1 mg/mL) (Sigma-Aldrich, St. Louis, MO, USA), 0.20 μL lysostaphin 10 mg/mL (final concentration 0.02 mg/mL) (ThermoFisher Scientific, USA), and 0.10 μL RNAse A 100 mg/mL (final concentration 0.1 mg/mL) (Qiagen, Hilden, Germany). The bacterial suspension from an overnight culture was centrifuged at 8000× *g* for 2 min, and the pelleted bacterial cells were resuspended in 100 μL of the pre-lysis buffer by pipetting up and down several times with a P200 pipette. The suspension was then incubated for 60 min. After incubation, 1.00 μL of Proteinase K (Sigma-Aldrich, St. Louis, MO, USA) was added as a final step in the pre-lysis stage. The sample was then ready for the first step of the Qiagen DNA extraction protocol as discussed in the previous section.

After DNA quantification by NanoDrop (ThermoFisher Scientific 1000 NanoDrop Spectrophotometer), the samples were sent to microbesNG in the UKfor WGS by Illumina next-generation sequencing (https://microbesng.co.uk, Birmingham, UK, accessed on 30 June 2021) [60]. The extracted DNA was prepared following the manufacturer’s protocol as described on the company’s website as follows: ILLUMINA SEQUENCING (SGS and EGS) Genomic DNA libraries are prepared using the Nextera XT Library Prep Kit (Illumina, San Diego, CA, USA) following the manufacturer’s protocol with the following modifications: input DNA is increased 2-fold, and PCR elongation time is increased to 45 s. DNA quantification and library preparation are carried out on a Hamilton Microlab STAR automated liquid handling system (Hamilton Bonaduz AG, Bonaduz, Switzerland). Pooled libraries are quantified using the Kapa Biosystems Library Quantification Kit for Illumina. Libraries are sequenced using Illumina sequencers (HiSeq/NovaSeq) using a 250 bp paired-end protocol. Reads are adapter trimmed using Trimmomatic 0.30 with a sliding window quality cutoff of Q15 [63]. De novo assembly is performed on samples using SPAdes version 3.7 [64], and contigs are annotated using Prokka 1.11 [65].

### 4.6. Bioinformatics Analysis

For visualization of the *K. pneumoniae* contigs as well as the investigation of main virulence genes SnapGene Viewer 4.1 (https://snapgene.com, accessed on 12 January 2021) software was used. Moreover, Artemis was used to identify *Klebsiella* capsule synthesis loci in the different isolates [66]. Basic Local Alignment Search Tool (BLAST, https://blast.ncbi.nlm.nih.gov/blast.cgi accessed on 22 September 2021) was used to identify the similarities between the loci against a reference *K. pneumoniae* RJF293 (GenBank accession number CP014008) (https://blast.ncbi.nlm.nih.gov/Blast.cgi, accessed on 3 June 2021) [67]. In addition, Genbank was used to search for similar DNA sequences or genes from the DNA database to the sequenced genomes of this study. Kaptive was used for locus typing and variant analysis on the pre-assembled sequences against a *K. pneumoniae* database (https://kaptive-web.erc.monash.edu, accessed on 13 September 2021) [9]. The *Klebsiella* capsule synthesis loci (*k-loci*) of each of the sequenced genomes were generated and were used for comparison between the different isolates (Supplementary Figures S3–S30). Kleborate v2.1.0 was used to investigate other virulence factors including yersiniabactin, colibactin, aerobactin, and salmochelin [68]. The ICEfinder v2.0 online tool was used to detect integrative and conjugative elements (https://bioinfo-mml.sjtu.edu.cn/ICEfinder/ICEfinder.html, accessed on 26 May 2021) [69]. Multilocus sequence typing (MLST) v2.0.9 was determined using the MLST tool in the Center for Genomic Epidemiology (CGE) (http://cge.cbs.dtu.dk/services, accessed on 14 June 2021). PlasmidFinder and ResFinder in the CGE website were used to identify plasmids and acquired antimicrobial resistance genes, respectively [70]. Additionally, the Comprehensive Antibiotic Resistance Database v3.2.8 (CARD) (https://card.mcmaster.ca/, accessed on 30 April 2021) was used to detect putative antimicrobial resistance genes using the Resistance Gene Identifier (RGI) tool v6.0.3. This tool identifies the antibiotic resistome(s) as well as point mutations within the resistance-conferring genes [71]. Furthermore, the phylogeny tool in the CGE server was used to investigate the genetic relatedness between the different sequences by identifying single nucleotide polymorphisms (SNPs) [72]. The tree files were visualized using Mega7 v7.0, which performs automatic sequence alignment and infers phylogenetic trees [73]. In addition, a combined phylogenetic tree of 131 isolates that includes the isolates in this study as well as published global isolates was created using R v4.3.2, a statistical programming software for comparison and visualization of genetic similarities or differences between the project’s isolates and the published ones [74]. iTOL v6, an online tool, was used to display, annotate, and manage the phylogenetic tree [75]. A whole-genome SNP alignment was generated using Snippy v4.4.5 [75,76], and the AE015929 genome was used as a reference. Then, iqtree v1.6.12, using model finder and ultrafast bootstrap [77], was used to produce a maximum likelihood phylogenetic tree. A phylogenetic tree of the whole-genome SNP was constructed and linked to the gene analysis heat map using the R platform. Lists of clinical isolate serial numbers of phylogenetic trees in Figure 4 and Figure 5 are presented in Supplementary Table S4. For statistical analysis, one-way ANOVA was used to analyze variations in bactericidal activity of human serum against *K. pneumoniae* clinical isolates using GraphPad Prism software (10.0.3). A *p*-value of <0.05 was considered as statistically significant. Observation tables are presented in Supplementary Table S3.

### 4.7. G. mellonella Virulence Assays

Virulence assays were performed using the larvae of *G. mellonella* as a model of infection. The protocol was modified from previous published studies [78]. To ensure quality, the larvae were manually picked through local beekeepers in Oman and were kept at room temperature (25 °C) in the dark, and wood shavings were provided as a food source. Stage 6 larvae, approximately 2–3 cm in size, were selected for the purpose of the experiment. For each isolate and the controls, replicates of 10 and 5 larvae were used, respectively. The larvae were distributed in Petri dishes lined with wood shavings before injecting them with bacteria the next day. On day 1, the selected isolates for the experiment were sub-cultured onto CLED agar plates (Oxoid, Basingstoke Hampshire, UK) from cryo-beads. On day 2, three to five colonies were picked up and resuspended in 10 mL of liquid broth then incubated overnight at 37 °C. On day 3, a 1:100 dilution (1 mL bacterial suspension + 9 mL liquid broth) was grown for 3–4 h. The bacterial suspension was then centrifuged, the supernatant discarded, and the pelleted bacteria was washed three times with sterile 1× phosphate-buffered saline (PBS) to remove excess salts (Invitrogen, Agawam, MA, USA). The bacterial pellet was then resuspended in PBS to an absorbance of 0.2 (OD_600_), which was repeated for each isolate. The absorbance was measured using a spectrophotometer (Eppendorf BioPhotometer Spectrophotometer UV/VIS, St. Louis, MO, USA), and the colony-forming unit (CFU) counts were determined for each sample. For the test larvae, 10 μL of each sample (undiluted 0.2 OD_600_ suspension) was injected into the larvae. For Control 1 larvae, PBS only was injected, and for Control 2 larvae, they were kept untouched. For Control 3 larvae, Kp 99 non-hypermucovirulent (negative for any capsular serotype) bacterial suspension was injected. All larvae were injected through the haemocoel via the rear left pro-leg using an insulin syringe. Post injection, the larvae were incubated at 37 °C and their mortality was assessed every 24 h for 5 days. Over the course of 5 days, the larvae were examined with forceps by flipping them onto their backs and checking if there are signs of motility. Live larvae will quickly flip back, whereas sick larvae will be slow, the exterior will be hardened and darkened, and they will usually die within the coming 1–2 days. Dead larvae will have a hard, dry, and very dark exterior and will shrink in size. The results were observed and noted, and survival graphs were generated using GraphPad Prism 10.0.3 software. Observation data are presented in Supplementary Table S2.

### 4.8. Serum Resistance Assay

The susceptibility of bacteria to human serum was investigated using the previously described method [79] with some modifications. On day 1, the selected isolates for the experiment were sub-cultured onto agar plates from cryo-beads as well as *K. pneumoniae* ATCC 1705 that was used as a positive susceptible control in this experiment. On day 2, 3–5 colonies were picked up and resuspended in 10 mL of liquid broth then incubated overnight at 37 °C. On day 3, the bacterial suspension was diluted to 2 × 10^6^ CFU/mL. Then, 25 µL of bacterial suspension and 75 µL of human serum (Sigma-Aldrich, St. Louis, MO, USA) were dispensed in microtitration trays and mixed then incubated at 37 °C. To assess viability, the samples were streaked onto CLED agar plates immediately at time zero, after 1 h, after 2 h, and finally after 3 h. Each isolate was tested 3 times. The streaked agar plates were incubated overnight. On day 4, CFU counts of each sample and the control at 4 different time points were recorded, and graphs were made using Graphpad Prism 10.0.3. (Supplementary Table S3 and Figures S6–S10).

## 5. Conclusions

In summary, to our knowledge, this is the first study in Oman to depict the molecular and genomic characteristics of hypervirulent *K. pneumoniae* isolates collected from all around the country, where analysis of WGS of the isolates showed a distinct pattern of *k-loci* depending on the particular STs, particularly ST-231 and ST-395, which are the two most prominent STs. Resistance to serum bactericidal activity and *G. mellonella* lethality by hypervirulent capsular serotypes K1 and K2 *K. pneumoniae* strains was demonstrated. The hvKp strains in this study are diverse in the phylogenetic origin, but the likelihood of transmission and future outbreaks is key to detect possible spread of hvKp isolates in healthcare settings via active surveillance.

## Figures and Tables

**Table 1:** Sequence types of local *K. pneumoniae* clinical isolates (*n* = 31).

Isolate (Kp)	Sequence Type	Percentage (%)
102, 106, 110, 112, 113, 114, 115, 116, 118, 125	231	32
103, 104, 108, 111,121, 123	395	19
124 and 128	23	6
105 and 117	405	6
100	13	1
101	45	1
107	1714	1
109	280	1
119	1710	1
120	147	1
122	37	1
126	881	1
127	11	1
83	14	1
129	86	1

**Table 2:** Source and molecular genotype of clinical hypervirulent *K. pneumoniae* isolates.

Isolate	Serotype	ST	Source	Cluster
Kp 83	K2	14	Urine	D
Kp 124	K1	23	Pus	C
Kp 125	K2	231	Urine	B
Kp 126	K2	881	Urine	A

**Table 3:** Antimicrobial resistance genotype and phenotype of hypervirulent *K. pneumoniae* isolates.

Strain Name, Serotype, and ST	Plasmid	Resistance Phenotype	Resistance Genotype	Resistance Mechanism	Drug Class
Kp 83 K2/ST-14	IncFIA(HI1) 204,189	*ESBL*	*tet (B)*, *tetR*	Antibiotic efflux, target alteration	Tetracycline
		AMP, TZP, FEP, CTX, IMP, CAZ, CIP			
	IncFIB(K) 208,191 bp		*catI*	Antibiotic inactivation	Phenicol
			*drfA12*	Antibiotic target replacement	Folate pathway antagonist
			*aadA2*	Antibiotic inactivation	Aminoglycoside
			*qacEdelta1*	Efflux	Disinfecting agents and intercalating dyes
			*sul1*	Target replacement	Sulfonamides
			*mphA*	Antibiotic inactivation	Macrolides
	IncFIB(pNDM-Mar) 372,826 bp		*OXA-1*	Antibiotic inactivation	Carbapenem, Cephalosporin
			*NDM-1*	Antibiotic inactivation	Carbapenem, Cephalosporin
			*QnrB1*	Target protection	Quinolones
			*catI*	Antibiotic inactivation	Phenicol
			*CTX-M-15*	Antibiotic inactivation	Cephalosporin
			*AAC(6′)-lb-cr6*	Antibiotic inactivation	Quinolones, Aminoglycoside
	IncR 68,649 bp		*tet(D)*	Efflux	Tetracycline
			*sul2*	Target replacement	Sulfonamides
			*drfA14*	Target replacement	Folate pathway antagonist
			*SHV-2*	Inactivation	Carbapenem, Cephalosporin
			*QnrS1*	Target protection	Quinolones
			*APH(6)-Id*	Inactivation	Aminoglycoside
Kp125 K2/ST-231	ColKP3 5095 bp	*KPC*	*OXA-181*	Antibiotic inactivation	Carbapenem, Cephalosporin
	IncFIB(pQil) 115,300 bp	AMP, TZP, FEP, CTX, CAZ	*KPC-3*	Antibiotic inactivation	Carbapenem, Cephalosporin
			*TEM-1*	Antibiotic inactivation	Carbapenem, Cephalosporin
			*APH(3′)-la*	Antibiotic inactivation	Aminoglycoside
	IncFII(pAMA1167-NDM-5) 175,879 bp		*sul1*	Target replacement	Sulfonamide
			*qacEdelta1*	Efflux	Disinfecting agents and intercalating dyes
			*aadA5*	Antibiotic inactivation	Aminoglycoside
			*NDM-5*	Antibiotic inactivation	Carbapenem, Cephalosporin
			*mphA*	Antibiotic inactivation	Macrolides
			*drA17*	Target replacement	Folate pathway antagonist
			*tetR*	Antibiotic efflux, target alteration	Tetracyclines
Kp124 K1/ST-23	IncFIB(pQil) 115,300 bp	*ESBL/KPC*	*KPC-3*	Antibiotic inactivation	Carbapenem, Cephalosporin
		AMP, TZP, FEP, CTX, FOX, CAZ			
Kp126 K2/ST-881	IncFIB(K) 208,191 bp	*ESBL*	*catI*	Antibiotic inactivation	Phenicol
		AMP, FEP, CTX, IMP, CAZ, CIP	*drfA12*	Antibiotic target replacement	Folate pathway antagonist
			*aadA2*	Antibiotic inactivation	Aminoglycoside
			*qacEdelta1*	Efflux	Disinfecting agents and intercalating dyes
			*sul1*	Target replacement	Sulfonamides
			*mphA*	Antibiotic inactivation	Macrolides

**Table 4:** Interpretive categories and zone diameter breakpoints according to CLSI guidelines.

Antibiotic	Abbreviation	Disk Content (μg)	Zone Diameter Breakpoints (mm)		
			Susceptible	Intermediate	Resistant
Ampicilin	AMP	10	≥17	14–16	≤13
Piperacillin-tazobactam	TZP	110	≥21	18–20	≤17
Cefepime	FEP	30	≥25	19–24	≤18
Cefotaxime	CTX	30	≥26	23–25	≤22
Cefoxitin	FOX	30	≥18	15–17	≤14
Ceftazidime	CAZ	30	≥21	18–20	≤17
Imipenem	IMP	10	≥23	20–22	≤19
Meropenem	MEM	10	≥23	20–22	≤18
Gentamicin	CN	30	≥15	13–14	≤12
Amikacin	AK	10	≥17	15–16	≤14
Ciprofloxacin	CIP	5	≥31	21–30	≤20

## Data Availability

All supporting data can be found in the Supplementary Files. All whole-genome sequencing data are deposited in Genbank at accession number PRJNA999478. The raw data supporting the conclusions of this article will be made available by the authors, without undue reservation.

## Supplementary Materials

The following supporting information can be downloaded at: https://www.mdpi.com/article/10.3390/ijms25031944/s1. Reference [80] is cited in the Supplementary Materials.
