# Supplementary material for: Hypervirulent Capsular Serotypes K1 and K2 Klebsiella pneumoniae Strains Demonstrate Resistance to Serum Bactericidal Activity and Galleria mellonella Lethality
Source: Int J Mol Sci. 2024 Feb 5;25(3):1944. doi: 10.3390/ijms25031944 (PMC10855873; doi:10.3390/ijms25031944)
Supplement: Supplementary file 1 [file ijms-25-01944-s001.zip › ijms-2835488-supplementary.pdf]

Supplementary Materials

1 Supplementary Figures

(A) PCR screening for capsular serotypes in clinical isolates (n=129)

A.1 Colony Polymerase Chain Reaction

Detection and characterization of hypermucovirulent capsular serotypes K1, K2, K20, K54 and K57

DNA was extracted using the boiling method or thermal lysis in preparation for PCR. Firstly, 30 µl of nuclease-free water (Invitrogen, USA) was used to re-suspend three to five colonies picked up from the agar plate. Secondly, the samples were heated for 10 minutes at 100 °C in a heat block (Eppendorf ThermoStat plus, Germany). Thirdly, the samples were centrifuged at 16000 *x g* for 1 minute and the supernatant was used as a template for PCR whilst the cell pellet was discarded.

The PCR assays were performed using GoTaq® G2 Green Master Mix (Promega Ltd., USA). The cycling conditions for the Go-Taq polymerase are listed in Table 2.2. The detection of the five *K. pneumoniae* hyper-virulent capsular serotypes was performed by the amplification of the capsular genes K1, K2, K20, K54 and K57. The conditions of the PCR cycles were set according to the size of the genes and the melting temperature of the primers.

The reaction mixture consisted of 12.5 µl of the GoTaq® G2 Green Master Mix, 2X, 1 µl forward primer, 1 µl reverse primer, 1 µl Dimethyl sulfoxide (DMSO) (Sigma-Aldrich, USA), 1.5 µl of the DNA template and finally nuclease-free water was added so that the total volume is 25 µl. DMSO enhances the annealing of primers to the template and therefore positively impacts the process of amplification. A positive control (when available, depending on the capsular serotype) was used as well as a negative control that consists of the reaction mixture without the DNA template. A thermocycler (Eppendorf master cycler®, MA, Germany) was used to perform the amplification. The annealing temperatures for each gene as they differ based on the melting temperature of the primers as well as the specific primers used for the detection of the 5 different capsular serotypes are listed in Supplementary Table 14.

A.2 Gel Electrophoresis

The gel was prepared using 1.4 g of low electroendosmosis (LE) agarose, 70 ml 1x Tris/Borate/EDTA (TBE) electrophoresis buffer (ThermoFisher Scientific, USA) and 4 µl MIDORI^{Green} Direct (NIPPON-genetics, Europe). GeneRuler 1 kb DNA Ladder (ThermoFisher Scientific, USA) was used to estimate the size of the DNA fragments. Finally, the bands were visualised using iBright™ CL1500 Imaging System (Invitrogen™, USA).

One hundred and twenty-six isolates (Kp 1 – Kp 123, Kp 127 – Kp 129) were screened for five hypervirulent capsular serotypes K1, K2, K20, K54 and K57 using conventional PCR amplification. Kp 124, Kp 125 and Kp126 were used as positive controls in this experiment as they were tested previously. Kp 124 is positive for K1, Kp 125 and Kp 126 are positive for K2 as shown in supplementary figure 1 (A, B and C). Further analysis of their hypervirulent nature including WGS, phylogeny and investigation of *k-loci* was continued in this study.

Moreover, Kp 83 was positive for capsular serotype 2 and none of the 129 isolates were positive for K20, K54 and K57. Faint bands in lanes 75, 78 and 80 were observed which might be non-specific bands. Therefore, to double check, PCR and gel electrophoresis were repeated for Kp 75, 78 and 80 with slight modifications including the addition of DMSO which resulted in the disappearing of the bands, therefore, these were excluded from WGS studies.

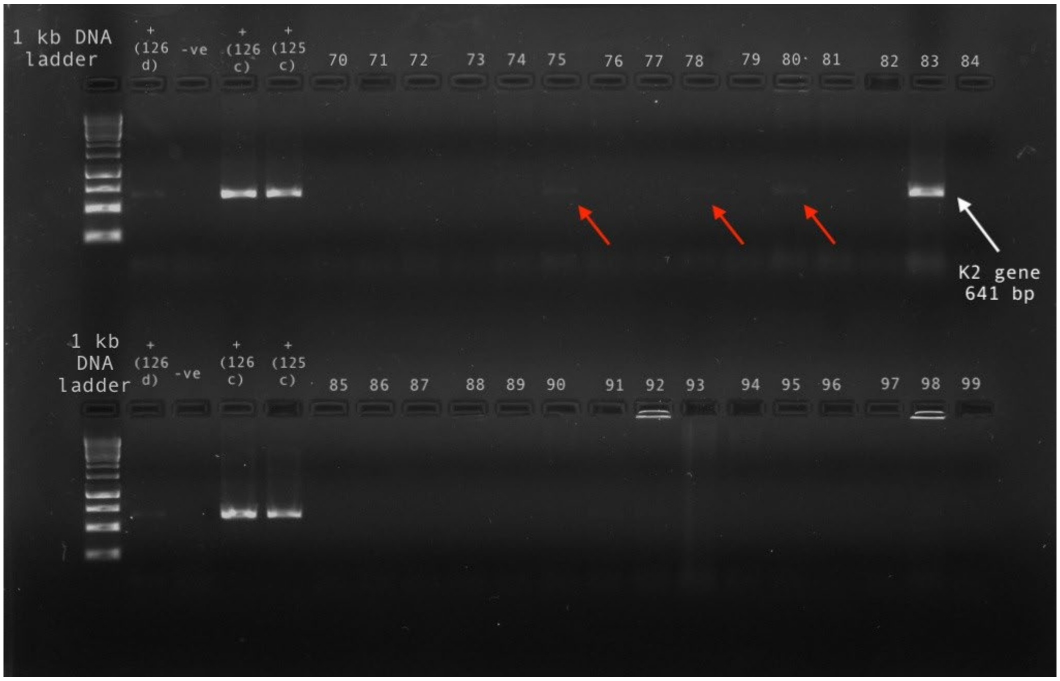

Supplementary Figure S1. Agarose gel electrophoresis of the K2 hypervirulent isolate Kp 83. Lane 1 is1 kb DNA ladder. Lane 2 is the positive control Kp 126 (DNA sample isolated in 2019), Lane 3 is the negative control. Lanes 4 and 5 are positive controls Kp 126 and Kp 125 (DNA extracted by boiling method from fresh overnight culture). Lanes 75, 78 and 80 isolates Kp 75, Kp 78 and Kp 80, respectively show very faint bands with an expected size of 641 bp (pointed at with red arrows). Lane 83 is isolate Kp 83, positive for K2 with an expected band size of 641 bp.

The gel electrophoresis images below show PCR products of isolates Kp 124, Kp 125 and Kp 126 for the hypervirulent capsular serotypes. Positive isolates that show dense and significant DNA bands were sent for WGS. Those isolates are Kp 124, Kp 125, Kp 126 and Kp 83.

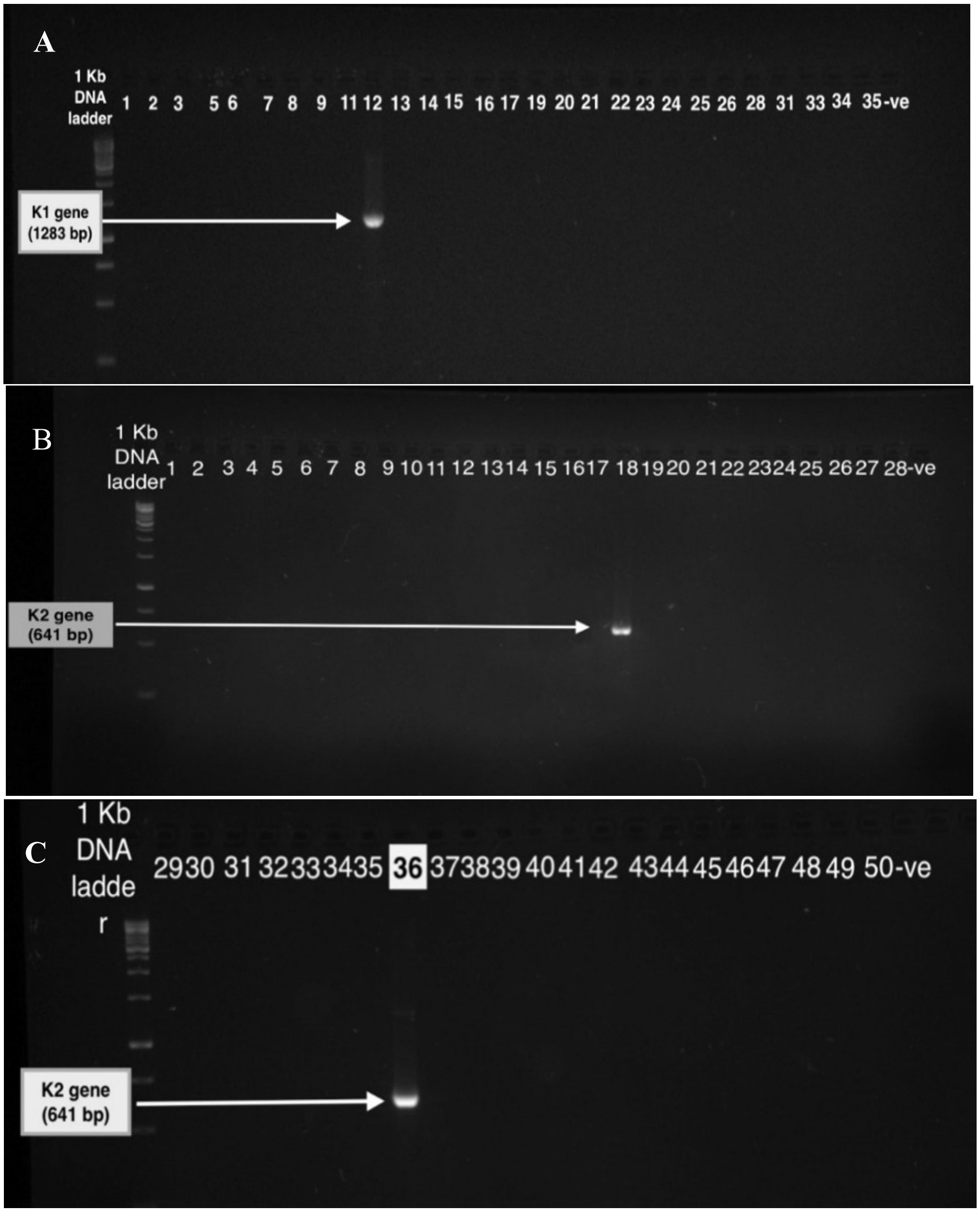

Supplementary Figure S2. Agarose gel electrophoresis of the K1 and K2 hypervirulent capsular serotypes. Lane 1 is the 1 kb DNA ladder in A, B and C. Lane 36 in A, lane 29 in B and lane 51 in C is the negative control. Lane 12 in A is isolate Kp 124, positive for K1 with an expected band size of 1238 bp. Lane 18 in B is isolate Kp 125, positive for K2 with an expected band size of 641 bp. Lane 36 in C is isolate Kp 126, positive for K2 with an expected band size 641 bp.

(B) Kaptive

Kaptive online tool was used to compare the *k-loci* of the isolates to known *K. pneumoniae* references in its database to analyse the similarities and variabilities between the isolates [1] (Wick et al., 2018). Thefigures generated by Kaptive represent the arrangement of different *k-loci* in each isolate and the genesthat make up these loci as well as percentage of similarity between the isolate to known references. Many of the isolates have similar Kaptive figures therefore only the four most common ones are represented in supplementary figures 27, 288, 29 and 30.

The Kaptive image below shows the *k-loci* genetic arrangement of isolates Kp 102, Kp 106, Kp 110, Kp 112, Kp 113, Kp 114, Kp 115, Kp 116, Kp 118 and Kp 125. The *k-loci* of these isolates are similar to a known reference, KL 51 in the Kaptive database. However, there is a missing part of the genetic sequence represented as a line (**____**) in the figure below as well as a missing gene, *wcoZ*, representedin gray.

Kaptive figures for all clinical isolates subjected to WGS

Kp 100

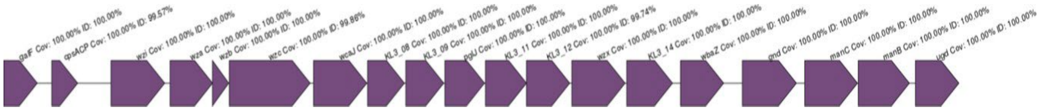

Supplementary Figure S3. *k-loci* gene arrangement in Kp 100 . The *k-loci* of the isolate is perfectly similar toa known reference, KL52. The match confidence is very good.

Kp 101

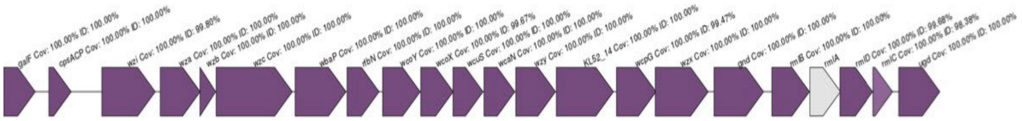

Supplementary Figure S4. *k-loci* gene arrangement in Kp 101. The *k-loci* of the isolate is perfectly similar toa known reference, KL52 except for 1 gene, *rmlA*, shown in gray that is very different. The match confidence is just good.

Kp 102

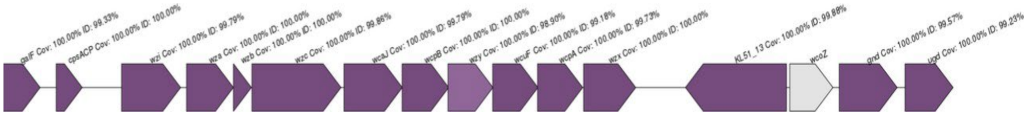

Supplementary Figure S5. *k-loci* gene arrangement in Kp 102. The *k-loci* of the isolate is similar to a known reference, KL51. However, there is a missing part of the genetic sequence represented as a line (__) in the figure above as well as a very different gene, *wcoZ*, represented in gray.

Kp 103

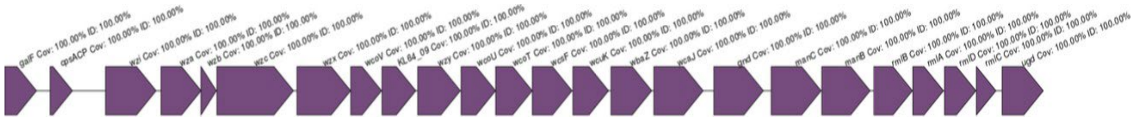

Supplementary Figure S6. *k-loci* gene arrangement in Kp 103. The *k-loci* of the isolate is perfectly similar toa known reference, KL64. The match confidence is very good.

Kp 104

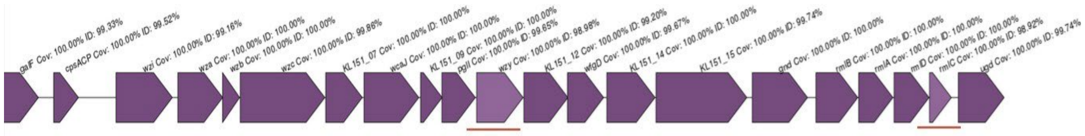

Supplementary Figure S7. *k-loci* gene arrangement in Kp 104. The *k-loci* of the isolate is similar to a known reference, KL64 except for 2 genes *wcoU & wcoT,* shown in gray that are very different. The match confidence is just good.

Kp 105

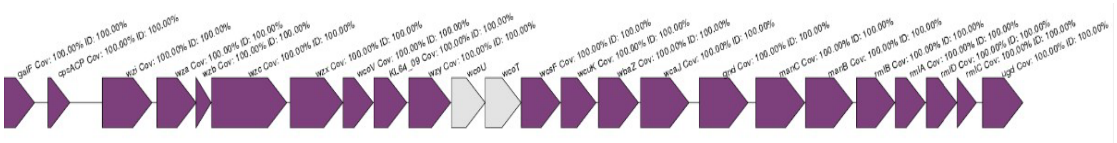

Supplementary Figure S8. *k-loci* gene arrangement in Kp 105. The *k-loci* of the isolate is very similar to a known reference, KL151 except for 2 genes that are a bit different as they are represented in light purple in the figure and underlined in red, *rmIC* and *wzy.*

Kp 106

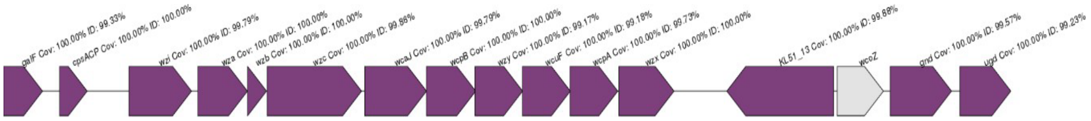

Supplementary Figure S9. *k-loci* gene arrangement in Kp 106. The *k-loci* of the isolate is similar to a known reference, KL51. However, there is a missing part of the genetic sequence represented as a line (__) in the figure above as well as a very different gene, *wcoZ*, represented in gray. The *k-loci* in this isolate is exactly identical to Kp 102.

Kp 107

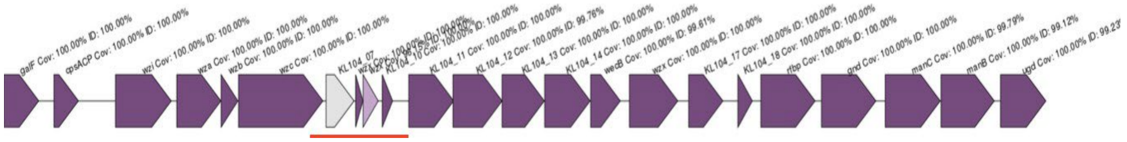

Supplementary Figure S10. *k-loci* gene arrangement in Kp 107. The *k-loci* of the isolate is similar to a known reference, KL104. The match confidence is good but the arrangement of genes in this k-loci is a bit different than other isolates. The light purple means low coverage or identity in gene *wzx* as well as there is a missing gene represented in gray, *KL104_7*.

Kp 108

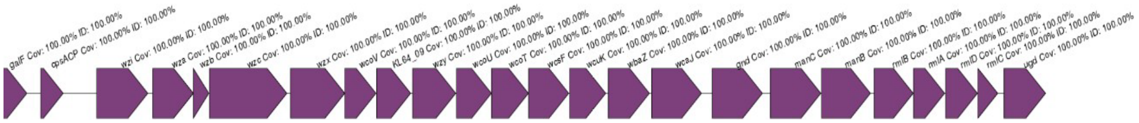

Supplementary Figure S11. *k-loci* gene arrangement in Kp 108. The *k-loci* of the isolate is similar to a known reference, KL64. The match confidence is very good. The *k-loci* in this isolate is exactly identical to Kp 103.

Kp 109

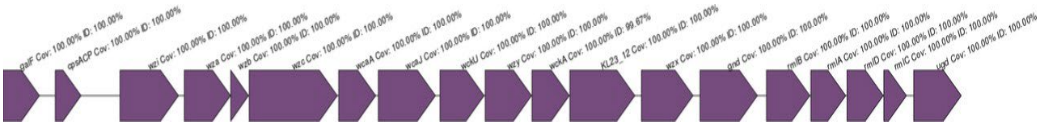

Supplementary Figure S12. *k-loci* gene arrangement in Kp 109. The *k-loci* of the isolate is similar to a known reference, KL23. The match confidence is very good.

Kp 110

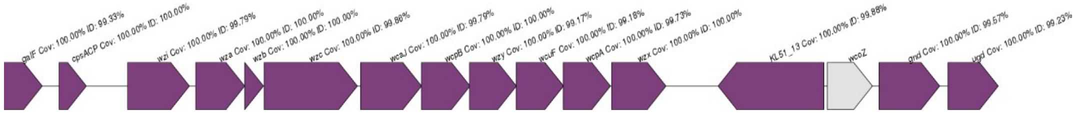

Supplementary Figure S13. *k-loci* gene arrangement in Kp 110. The *k-loci* of the isolate is similar to a known reference, KL51. However, there is a missing part of the genetic sequence represented as a line (__) in the figure above as well as a very different gene, *wcoZ*, represented in gray. The *k-loci* in this isolate is exactly identical to Kp 102 & 106.

Kp 111

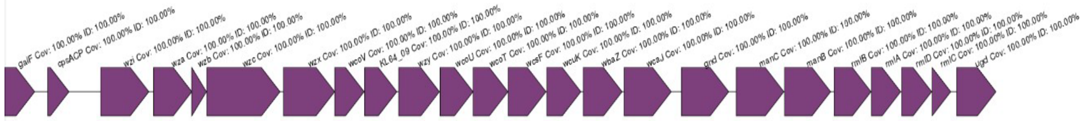

Supplementary Figure S14. *k-loci* gene arrangement in Kp 111. The *k-loci* of the isolate is similar to a known reference, KL64. The match confidence is very good. The *k-loci* in this isolate is exactly identical to Kp 108 & 103.

Kp 112

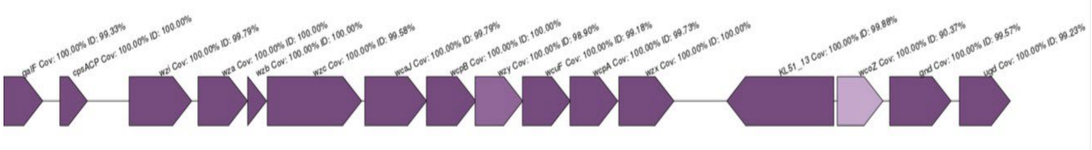

Supplementary Figure S15. *k-loci* gene arrangement in Kp 112. The *k-loci* of the isolate is similar to a known reference, KL51. However, there is a missing part of the genetic sequence represented as a line (__) in the figure above as well as a somewhat different gene, *wcoZ*, represented in light purple. The *k-loci* in this isolate is exactly identical to Kp 102 ,106 & 110.

Kp 113

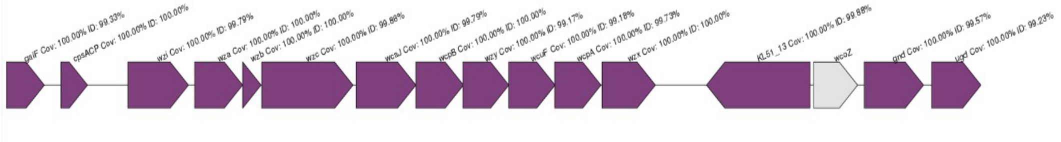

Supplementary Figure S16. *k-loci* gene arrangement in Kp 113. The *k-loci* of the isolate is similar to a known reference, KL51. However, there is a missing part of the genetic sequence represented as a line (__) in the figure above as well as a very different gene, *wcoZ*, represented in gray. The *k-loci* in this isolate is exactly identical to Kp 102, 106, 110 & 112.

Kp 114

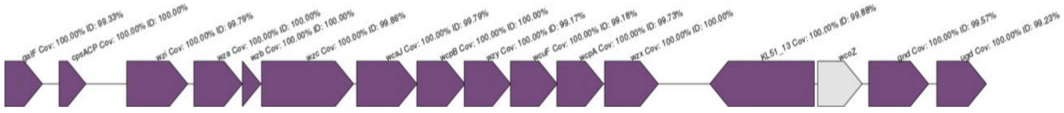

Supplementary Figure S17. *k-loci* gene arrangement in Kp 114. The *k-loci* of the isolate is similar to a known reference, KL51. However, there is a missing part of the genetic sequence represented as a line (__) in the figure above as well as a very different gene, *wcoZ*, represented in gray. The *k-loci* in this isolate is exactly identical to Kp 102, 106, 110, 112 & 113.

Kp 115

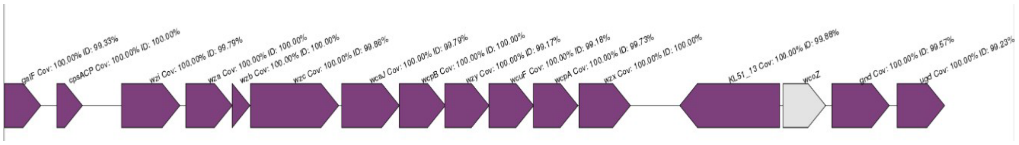

Supplementary Figure S18. *k-loci* gene arrangement in Kp 115. The *k-loci* of the isolate is similar to a known reference, KL51. However, there is a missing part of the genetic sequence represented as a line (__) in the figure above as well as a very different gene, *wcoZ*, represented in gray. The *k-loci* in this isolate is exactly identical to Kp 102, 106, 110, 112, 113 & 114.

Kp 116

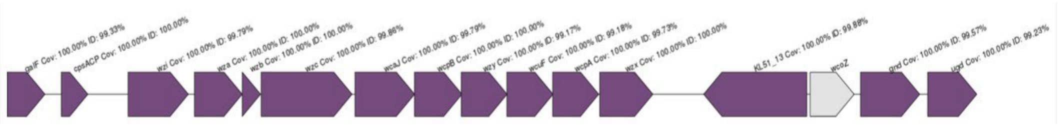

Supplementary Figure S19. *k-loci* gene arrangement in Kp 116. The *k-loci* of the isolate is similar to a known reference, KL51. This genetic composition is unique with a very different gene, *wcoZ*, represented in gray. The*k-loci* in this isolate is exactly identical to Kp 102, 106, 110, 112, 113, 114 & 115.

Kp 117

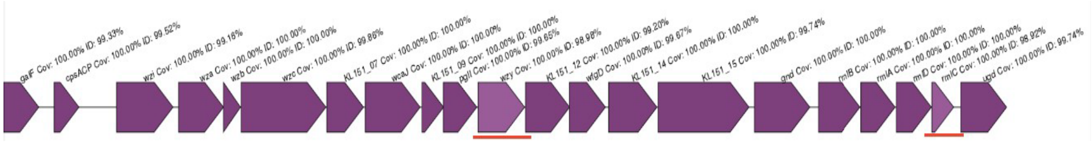

Supplementary Figure S20. *k-loci* gene arrangement in Kp 117. The *k-loci* of the isolate is very similar to a known reference, KL151 except for 2 genes that are a bit different as they are represented in light purple in the figure and underlined in red, *rmIC* and *wzy.* The *k-loci* in this isolate is exactly identical to Kp 105.

Kp 118

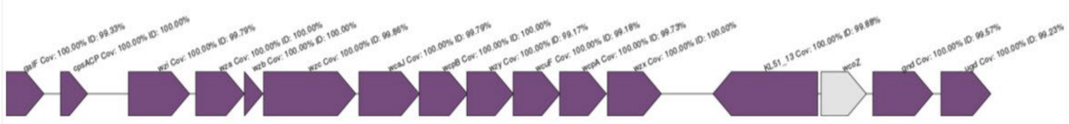

Supplementary Figure S21. *k-loci* gene arrangement in Kp 118. The *k-loci* of the isolate is similar to a known reference, KL51. However, there is a missing part of the genetic sequence represented as a line (__) in the figure above as well as a very different gene, *wcoZ*, represented in gray. The *k-loci* in this isolate is exactly identical to Kp 102, 106, 110, 112, 113, 114, & 115.

Kp 119

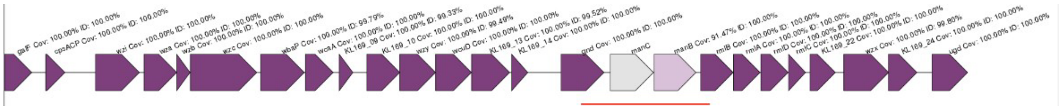

Supplementary Figure S22. *k-loci* gene arrangement in Kp 119. The *k-loci* of the isolate is similar to a known reference, KL169. The match confidence is not very good as the genetic sequence of this isolate was broken into 7 pieces hence, the assembly was poor. The underlined genes are inserted in a different position than the rest of the isolates, they are usually in the end. The gene that is absent or very different represented in gray is *manC* and the light purple which is a little different than the reference is *manB*.

Kp 120

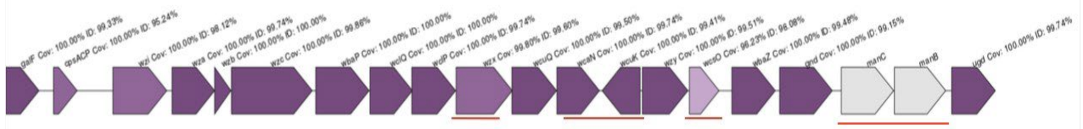

Supplementary Figure S23. *k-loci* gene arrangement in Kp 120. The *k-loci* of the isolate is similar to a known reference, KL64. The match confidence is not very good. The *k-loci* in this isolate is somewhat similar to Kp 103, 108 & 111. However, the gene *wzx* is not very similar as it is in light purple, gene *wcsD* is quite different as it is represented in lavender as well as the *manB & manC* genes are absent. Also, the gene wcuZ is inserted in reverse.

Kp 121

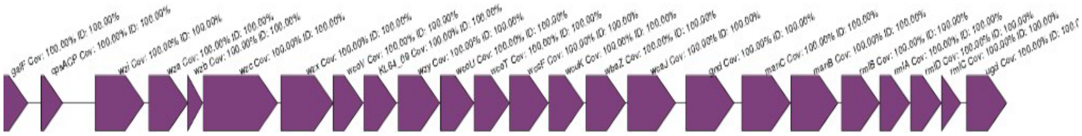

Supplementary Figure S24. *k-loci* gene arrangement in Kp 121. The *k-loci* of the isolate is similar to a known reference, KL64. The match confidence is very good. The *k-loci* in this isolate is exactly identical to Kp 103, 108 & 111.

Kp 122

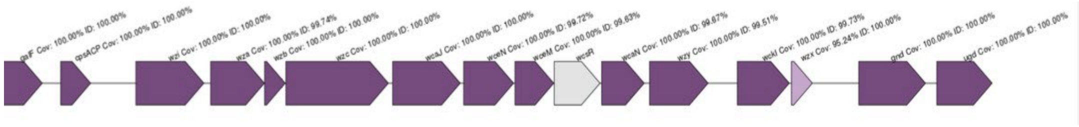

Supplementary Figure S25. *k-loci* gene arrangement in Kp 122. The *k-loci* of the isolate is similar to a known reference, KL15. The match confidence is not very good. There are a few gaps in the loci as well as a missing gene, *wcsR* and a gene with low coverage in lavender, *wzx*. This isolate has a *k-loci* that is very different than other isolates.

Kp 123

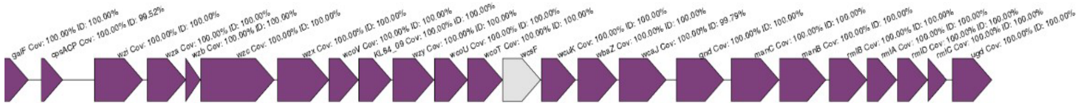

Supplementary Figure S26. *k-loci* gene arrangement in Kp 123. The *k-loci* of the isolate is similar to a known reference, KL64. The match confidence is good except for *wcsF* which is represented in gray, it might be absent or completely different than the reference. The *k-loci* in this isolate is exactly identical to Kp 103, 108 & 111.

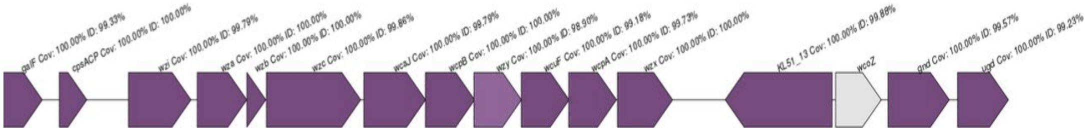

Supplementary Figure S27. *k-loci* gene arrangement of isolates Kp 102, Kp 106, Kp 110, Kp 112, Kp 113, Kp 114, Kp 115, Kp 116, Kp 118 and Kp 125.

The Kaptive image below shows the *k-loci* genetic arrangement of isolates Kp 103, Kp 104, Kp 108, Kp 111, Kp 120, Kp 121 and Kp 123. The *k-loci* of these isolates are similar to a known reference, KL 64 in the Kaptive database. The match confidence is very good.

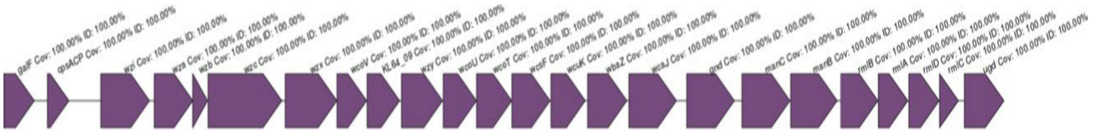

Supplementary Figure S28. *k-loci* gene arrangement of isolates Kp 103, Kp 104, Kp 108, Kp 111, Kp 120, Kp 121 and Kp 123.

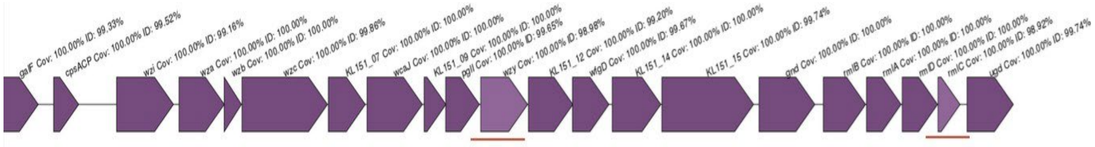

Supplementary Figure S29. *k-loci* gene arrangement of isolates Kp 105 and Kp 117. The *k-loci* of the isolates are very similar to a known reference, KL151 except for 2 genes that are a bit different as they are represented in light purple in the figure and underlined in red, *rmlC* and *wzy.*

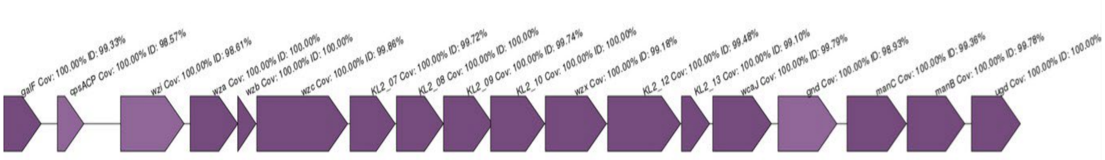

Supplementary Figure S30. *k-loci* gene arrangement of isolates Kp 83, Kp 126 and Kp 129. The Kaptive image below shows the *k-loci* genetic arrangement of isolates Kp 83, Kp 126 and Kp 129. The *k-loci* of these isolate are similar to a known reference, KL2. The match confidence is very high.

(C) Plasmid analysis

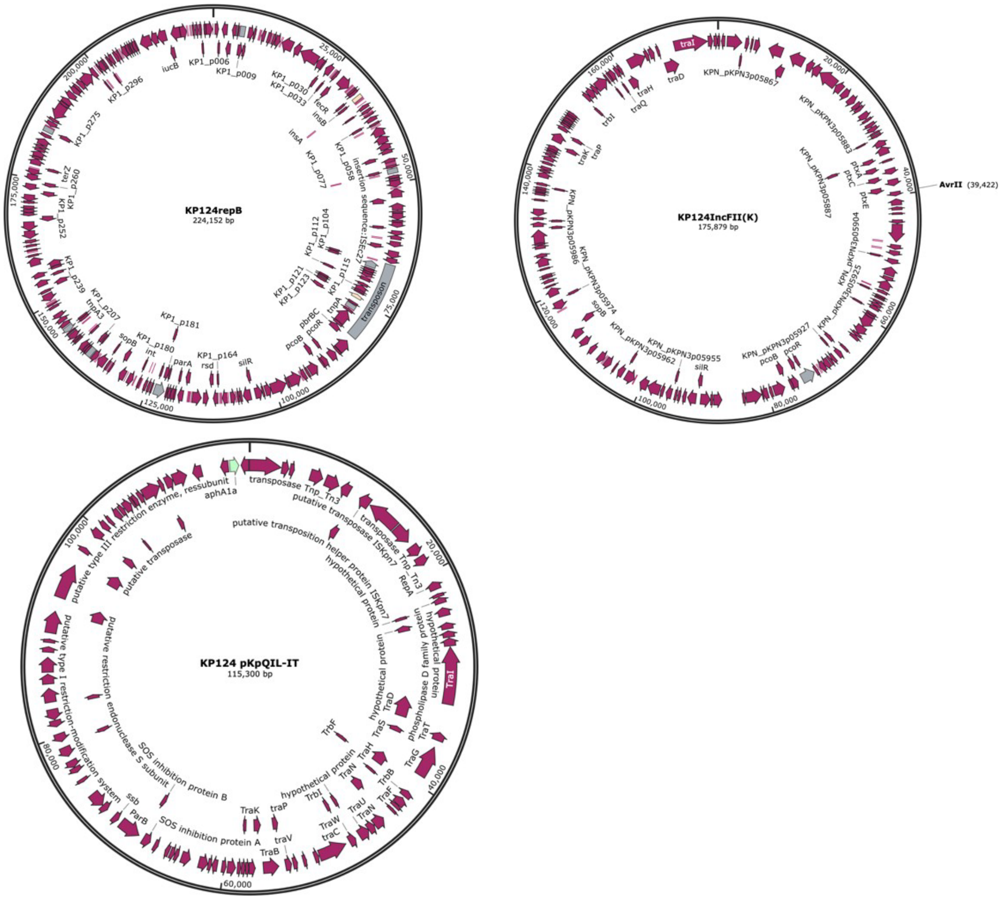

Supplementary Figure S31. Acquired plasmids carrying antibiotic resistance genes in KP124 K1/ST-23. The Open reading frames (ORFs) are indicated in purple arrows, the name and the size of each plasmid is indicated in bps is indicated inside each circular genome.

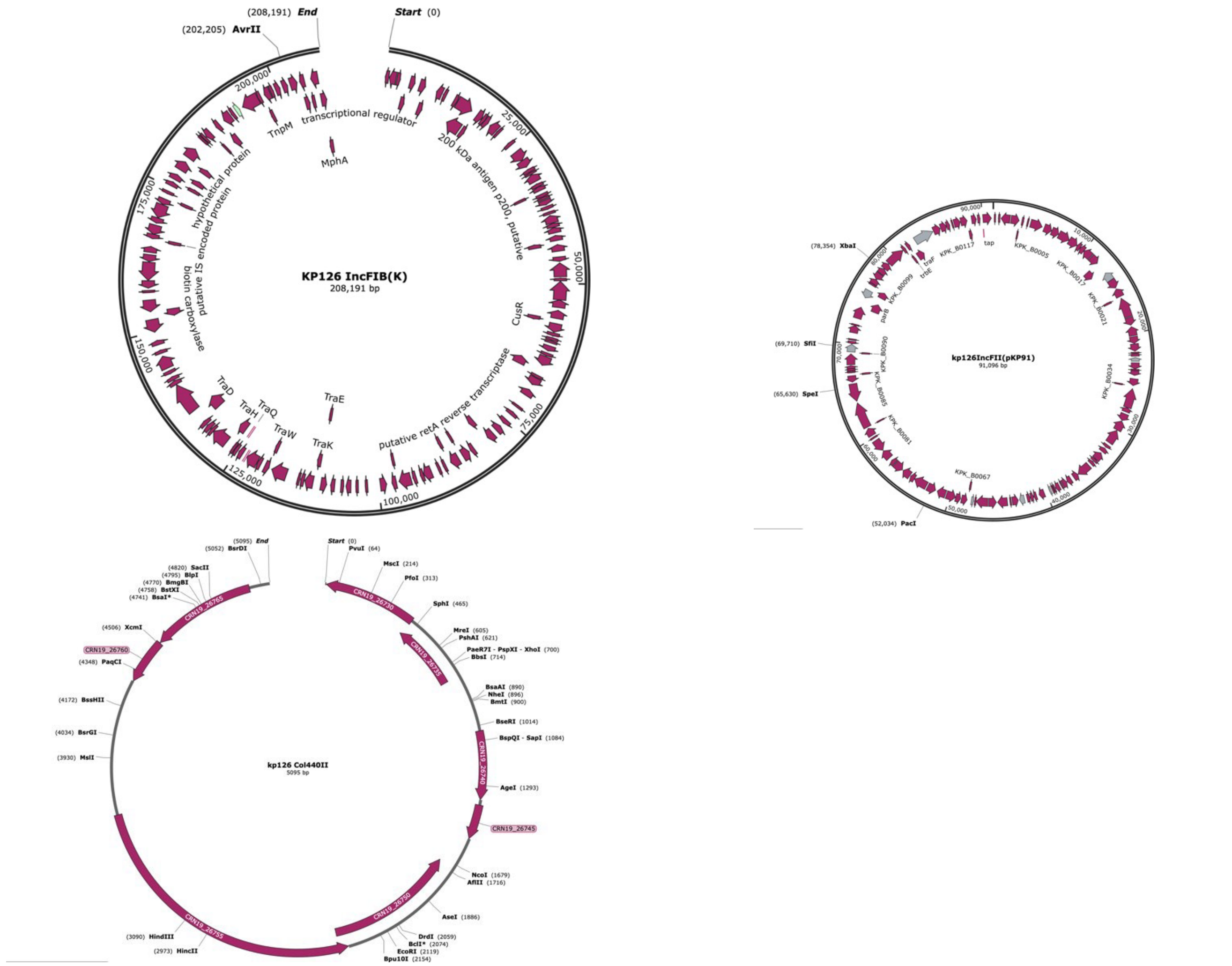

Supplementary Figure S32. Acquired plasmids carrying antibiotic resistance genes in KP126 K2/ST-881. The Open reading frames(ORFs) are indicated in purple arrows, the name and the size of each plasmid is indicated in bps is indicated inside each circular genome.

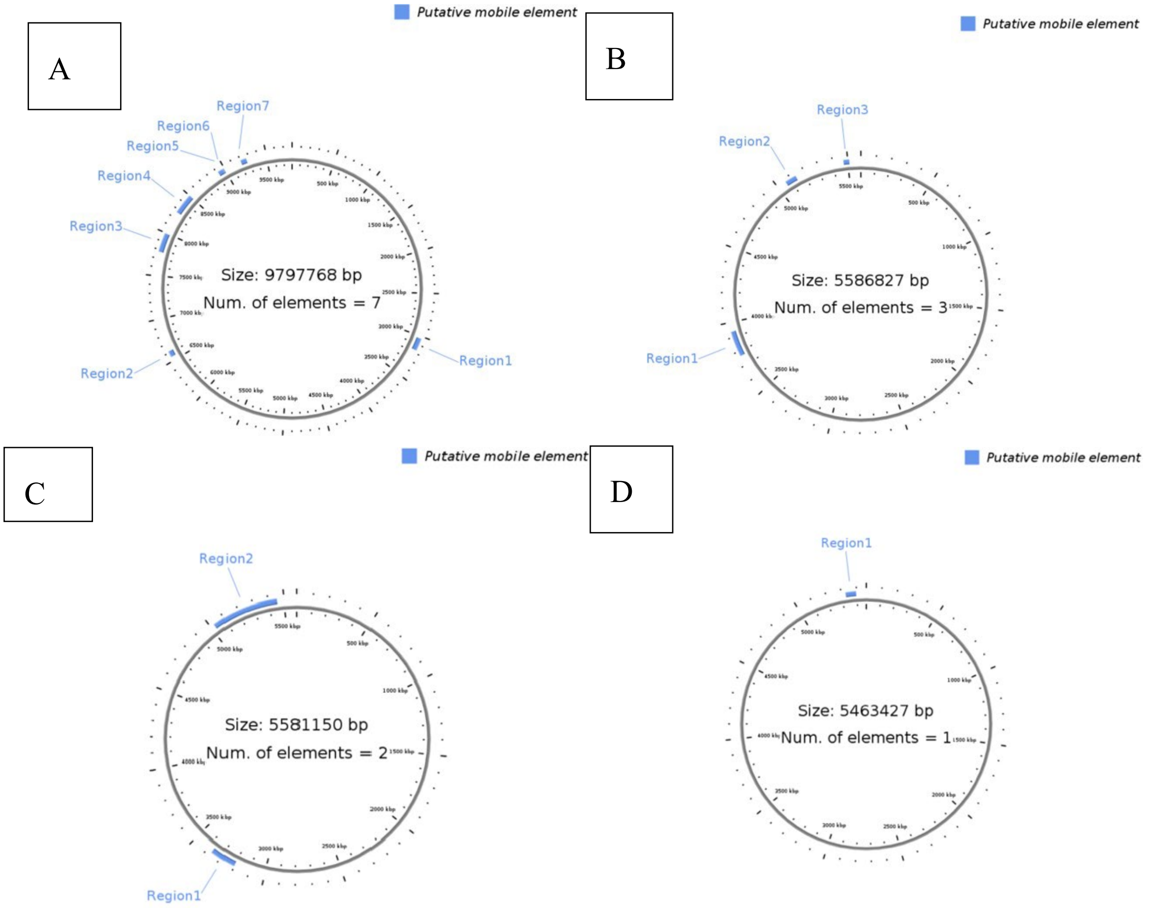

Supplementary Figure S33. Putative integrative and conjugative elements (ICEs) in (A) KP83 K2/ST-14, (B) KP124 K1/ST-23, (C) KP125 K2/ST231, and (D) KP126 K2/ST-881. The ICEs region in the genome is indicated in blue boxes, the size in bps and the number of elements are indicated inside each circular genome.

Supplementary Materials

1 Supplementary Tables

(A) List of clinical isolates

Supplementary Table S1. List of *K. pneumoniae* isolates (n=129).

Isolate	Year of isolation	Specimen/ Source	Place of collection
Kp 1	2018	Blood culture	CPHL
Kp 2	2018	Urine	CPHL
Kp 3	2018	Urine	CPHL
Kp 4	2018	Sputum	CPHL
Kp 5	2018	Sputum	CPHL
Kp 6	2018	Urine	CPHL
Kp 7	2018	Sputum	CPHL
Kp 8	2018	Wound	CPHL
Kp 9	2018	Blood culture	CPHL
Kp 10	2018	Tracheal aspirate	CPHL
Kp 11	2020	Tracheal aspirate	CPHL
Kp 12	2020	Blood culture	CPHL
Kp 13	2020	Tracheal aspirate	CPHL
Kp 14	2020	Urine	CPHL
Kp 15	2020	Urine	CPHL
Kp 16	2020	Urine	CPHL
Kp 17	2020	Urine	CPHL
Kp 18	2020	Urine	CPHL
Kp 19	2020	Urine	CPHL
Kp 20	2020	Tracheal aspirate	CPHL
Kp 21	2016	Blood culture	CPHL
Kp 22	2016	Blood culture	CPHL
Kp 23	2016	Sputum	CPHL
Kp 24	2016	Sputum	CPHL

Kp 25	2016	Blood culture	CPHL
Kp 26	2016	Wound	CPHL
Kp 27	2016	Wound	CPHL
Kp 28	2016	Urine	CPHL
Kp 29	2016	Blood culture	CPHL
Kp 30	2016	Urine	CPHL
Kp 31	2016	Wound	CPHL
Kp 32	2020	Blood culture	CPHL
Kp 33	2020	Tracheal aspirate	CPHL
Kp 34	2020	Tracheal aspirate	CPHL
Kp 35	2020	Blood culture	CPHL
Kp 36	2020	Blood culture	CPHL
Kp 37	2020	Tracheal aspirate	CPHL
Kp 38	2020	Tracheal aspirate	CPHL
Kp 39	2020	Urine	CPHL
Kp 40	2020	Tracheal aspirate	CPHL
Kp 41	2020	Sputum	CPHL
Kp 42	2015	Tracheal aspirate	CPHL
Kp 43	2015	Tracheal aspirate	CPHL
Kp 44	2015	Urine	CPHL
Kp 45	2015	Urine	CPHL
Kp 46	2015	Tracheal aspirate	CPHL
Kp 47	2015	Tracheal aspirate	CPHL
Kp 48	2015	Pus	CPHL
Kp 49	2015	Urine	CPHL
Kp 50	2019	Sputum	CPHL
Kp 51	2019	Blood culture	CPHL
Kp 52	2019	Urine	CPHL
Kp 53	2019	Urine	CPHL
Kp 54	2019	Tracheal aspirate	CPHL
Kp 55	2019	Sputum	CPHL

Kp 56	2019	Tracheal aspirate	CPHL
Kp 57	2019	Tracheal aspirate	CPHL
Kp 58	2019	Urine	CPHL
Kp 59	2019	Tracheal aspirate	CPHL
Kp 60	2017	Wound	CPHL
Kp 61	2017	Tracheal aspirate	CPHL
Kp 62	2017	Blood culture	CPHL
Kp 63	2017	Blood culture	CPHL
Kp 64	2017	Blood culture	CPHL
Kp 65	2017	Blood culture	CPHL
Kp 66	2017	Sputum	CPHL
Kp 67	2017	Wound	CPHL
Kp 68	2017	Sputum	CPHL
Kp 69	2017	Sputum	CPHL
Kp 70	2019	Urine	CPHL
Kp 71	2019	Tracheal aspirate	CPHL
Kp 72	2019	Sputum	CPHL
Kp 73	2019	Blood culture	CPHL
Kp 74	2019	Blood culture	CPHL
Kp 75	2019	Blood culture	CPHL
Kp 76	2019	Blood culture	CPHL
Kp 77	2019	Blood culture	CPHL
Kp 78	2019	Blood culture	CPHL
Kp 79	2019	Blood culture	CPHL
Kp 80	2017	Urine	CPHL
Kp 81	2017	Tracheal aspirate	CPHL
Kp 82	2017	Urine	CPHL
Kp 83	2017	Urine	CPHL
Kp 84	2017	Sputum	CPHL
Kp 85	2017	Urine	CPHL
Kp 86	2017	Urine	CPHL
Kp 87	2017	Urine	CPHL

Kp 88	2017	Urine	CPHL
Kp 89	2017	Urine	CPHL
Kp 90	2018	Blood culture	CPHL
Kp 91	2018	Urine	CPHL
Kp 92	2018	Blood culture	CPHL
Kp 93	2018	Urine	CPHL
Kp 94	2018	Urine	CPHL
Kp 95	2018	Tracheal aspirate	CPHL
Kp 96	2018	Urine	CPHL
Kp 97	2018	Blood culture	CPHL
Kp 98	2018	Blood culture	CPHL
Kp 99	2018	Blood culture	CPHL
Kp 100	2019	Urine	SQUH
Kp 101	2019	Urine	SQUH
Kp 102	2019	Urine	SQUH
Kp 103	2019	Wound	SQUH
Kp 104	2019	Wound	SQUH
Kp 105	2019	Blood culture	SQUH
Kp 106	2019	Tracheal aspirate	SQUH
Kp 107	2019	Peritoneal fluid	SQUH
Kp 108	2019	Tracheal aspirate	SQUH
Kp 109	2019	Urine	SQUH
Kp 110	2019	Wound	SQUH
Kp 111	2019	Urine	SQUH
Kp 112	2019	Urine	SQUH
Kp 113	2019	Urine	SQUH
Kp 114	2019	Tracheal aspirate	SQUH
Kp 115	2019	Tracheal aspirate	SQUH
Kp 116	2019	Wound	SQUH
Kp 117	2019	Tracheal aspirate	SQUH
Kp 118	2019	Catheter urine	SQUH

Kp 119	2019	Urine	SQUH
Kp 120	2019	Urine	SQUH
Kp 121	2019	Sputum	SQUH
Kp 122	2019	Tracheal aspirate	SQUH
Kp 123	2019	Urine	SQUH
Kp 124	2019	Pus	SQUH
Kp 125	2019	Urine	SQUH
Kp 126	2019	Urine	SQUH
Kp 127	2021	Blood culture	SQUH
Kp 128	2021	Blood culture	SQUH
Kp 129	2021	Blood culture	SQUH

(B) *Galleria Mellonella* virulence assays

Supplementary Table S2. Virulence assay observation table (Replicates 1, 2 and 3).

	Day 1	Day 2	Day 3	Day 4	Day 5
**Control 1**	All alive	All alive	All alive	All dead	All dead
	All alive	4 alive, 1 dead	4 alive, 1 dead	3 alive, 2 dead	3 alive, 2 dead
	All alive	All alive	All alive	All alive	All alive
**Control 2**	All alive	All alive	All alive	All dead	All dead
	All alive	All alive	All alive	All alive	All alive
**Control 3**	2 alive, 3 dead	2 alive, 3 dead	2 alive, 3 dead	2 alive, 3 dead	2 alive, 3 dead
	1 dead, 4 alive	1 dead, 4 alive	1 dead, 4 alive	2 dead, 3 alive	2 dead, 3 alive
**Isolate 12**	6 dead, 4 alive	7 dead, 3 alive	7 dead, 3 alive	7 dead, 3 alive	7 dead, 3 alive
	All dead	All dead	All dead	All dead	All dead
	5 alive, 5 dead	3 alive, 7 dead	3 alive, 7 dead	2 alive, 8 dead	2 alive, 8 dead
**Isolate 18**	4 alive, 6 dead	2 alive, 8 dead	1 alive, 9 dead	All dead	All dead
	2 alive, 8 dead	2 alive, 8 dead	2 alive, 8 dead	2 alive, 8 dead	2 alive, 8 dead
	4 alive, 6 dead	3 alive, 7 dead	3 alive, 7 dead	2 alive, 8 dead	1 alive, 9 dead
**Isolate 36**	8 alive, 2 dead	8 alive, 2 dead	7 alive, 3 dead	5 alive, 5 dead	3 alive, 7 dead
	4 alive, 6 dead	3 alive, 6 dead	2 alive, 8 dead	1 alive, 9 dead	1 alive, 9 dead
	5 alive, 5 dead	5 alive, 5 dead	4 alive, 6 dead	4 alive, 6 dead	3 alive, 7 dead
**Isolate 84**	6 alive, 4 dead	4 alive, 6 dead	4 alive, 6 dead	2 alive, 8 dead	All dead
	2 alive, 8 dead	1 alive, 9 dead	1 alive, 9 dead	1 alive, 9 dead	1 alive, 9 dead
	All dead	All dead	All dead	All dead	All dead

(C) Human serum resistance assays

Supplementary Table S3. Serum resistance assay observation table.

Time	Isolate	Replicate A	Replicate B	Replicate C
**0 hour**	**Control**	115	63	55
**1 hour**		0	0	0
**2 hour**		0	0	0
**3 hour**		0	0	0
**0 hour**	**Kp 124**	10	16	43
**1 hour**		19	11	23
**2 hour**		18	8	20
**3 hour**		10	16	43
**0 hour**	**Kp 125**	181	136	68
**1 hour**		118	101	59
**2 hour**		200	222	129
**3 hour**		238	251	155
**0 hour**	**Kp 83**	63	27	20
**1 hour**		48	18	5
**2 hour**		12	6	3
**3 hour**		41	90	39

**0 hour**	**Kp 126**	160	133	236
**1 hour**		70	166	323
**2 hour**		118	82	174
**3 hour**		249	266	344

(D) Phylogenetic tree serial numbers of the study isolates

Supplementary Table S4. Clinical isolate serial numbers of phylogenetic tree in Figures 4 and 5.

Isolate number	Serial number
Kp 100	30121wA1_4
Kp 101	30121wA2_21
Kp 102	30121wB1_5
Kp 103	30121wB2_22
Kp 104	30121wA3_41
Kp 105	30121wB3_42
Kp 106	30121wC1_6
Kp 107	30121wC_25
Kp 108	30121wC_43
Kp 109	30121wD2_27
Kp 110	30121wD1_7
Kp 111	30121wD3_44
Kp 112	30121wE2_28
Kp 113	30121wE1_10
Kp 114	30121wE3_45
Kp 115	30121wF1_11
Kp 116	30121wF2_30
Kp 117	30121wF3_46
Kp 118	30121wG1_15
Kp 119	30121wG2_37
Kp 120	30121wG3_49
Kp 121	30121wH1_16
Kp 122	30121wH2_40
Kp 123	30121wH3_50
Kp 124	34548_12
Kp 125	34549_18
Kp 126	34550_36
Kp 127	34551_KP1
Kp 128	34552_KP2
Kp 129	34554_KP4
Kp 83	34553_KP3

(E) PCR assays

Supplementary Table S5. Colony PCR cycling conditions.

Stage	Temperature (°C)	Time	Cycles
Initialization	95	2 min	1
Denaturation	95	30 s	30
Annealing	variable	30 s	30
Extension	72	1 min per kb	30
Final extension	72	10 min	1
Hold	4		

Supplementary Table S6. List of PCR primer names, nucleotide sequences and annealing temperatures used in this study [2] (Xu, et al., 2017) .

Primer	Nucleotide sequence (5' -3')	Target	Expected size (bp)	Annealing temperature
magAF	GGTGCTCTTTACATCATTGC	K1	1283	58
magAR	GCAATGGCCATTTGCGTTAG			
wzy-K2F	GACCCGATATTCATACTTGACAGAG	K2	641	59
wzy-K2R	CCTGAAGTAAAATCGTAAATAGATGGC			
wzy-K20F	CGGTGCTACAGTGCATCATT	K20	741	53
wzy-K20R	GTTATACGATGCTCAGTCGC			
wzx-K54F	CATTAGCTCAGTGGTTGGCT	K54	881	52
wzx-K54R	GCTTGAAAACACCATAGCAG			
wzy-K57F	CTCAGGGCTAGAAGTGTCAT	K577	1037	58
wzx-K57R	CACTAACCCAGAAAGTCGAG			

Supplementary Table S7. Whole Genome Sequencing assembly data.

Sample id	# contigs (>= 0 bp)	# contigs (>= 1000 bp)	Total length (>= 0 bp)	Total length (>= 1000 bp)	# contigs	Largest contig	Total length	GC (%)	N50	N75	L50	L75	# N's per 100 kbp
34551_KP1	193	81	6053695	6020204	98	392411	6031950	56.63	195291	81844	11	24	0
34552_KP2	135	29	5671209	5632539	48	1079067	5645956	57.1	327304	238084	5	10	0
34553_KP3	448	258	9797768	9717439	315	423441	9758878	49.74	162064	73567	19	42	0
34554_KP4	108	35	5497672	5478888	37	572124	5480102	57.2	362153	247136	6	11	0
34548_12	192	130	5586827	5564299	140	264131	5572070	57.27	92449	42287	22	45	0
34549_18	110	58	5581150	5562044	67	1358108	5569468	57.12	355721	192308	4	9	0
34550_36	95	30	5463427	5446376	32	1522822	5447756	57.22	368795	289790	4	8	0
30121wA1_4	94	50	5756197	5742785	53	782668	5744772	56.74	419303	225410	5	10	0
30121wA2_21	156	57	5565747	5538270	64	607806	5543631	57.21	201678	133088	8	17	0
30121wA3_41	156	82	5885962	5860274	92	487142	5867169	56.57	142657	87033	12	25	0
30121wB1_5	147	67	5575295	5549057	78	567142	5556797	57.08	333829	144456	7	13	0
30121wB2_22	137	76	5501737	5483479	79	592150	5485792	57.19	197807	94980	10	21	0
30121wB3_42	114	45	5526667	5502286	57	783253	5511183	57.13	314823	139886	6	13	0
30121wC1_6	153	62	5596162	5562522	70	703565	5568762	57.1	301509	133177	7	13	0
30121wC2_25	91	35	5414163	5397667	38	627524	5399818	57.28	349817	170297	6	11	0
30121wC3_43	150	71	5546216	5521058	78	546961	5525883	57.08	230264	115681	9	17	0
30121wD1_7	139	61	5576390	5548183	69	696179	5554043	57.11	266959	144456	7	14	0
30121wD2_27	152	51	5527492	5496481	65	733509	5505576	57.26	292121	214896	6	12	0
30121wD3_44	159	73	5658842	5632215	83	780830	5638661	57.04	214134	95942	10	20	0
30121wE1_10	174	79	5551262	5516312	96	593340	5529527	57.05	306551	146426	7	14	0
30121wE2_28	119	64	5563947	5541131	78	936167	5551712	57.13	338602	144456	6	12	0
30121wE3_45	133	64	5517623	5492911	72	601014	5499212	57.13	355554	144456	7	12	0
30121wF1_11	169	74	5576075	5536919	94	630146	5550874	57.1	266959	131440	8	15	0
30121wF2_30	114	57	5582020	5563700	63	942792	5568129	57.1	387749	144456	4	10	0
30121wF3_46	229	44	5576334	5504095	58	1392436	5513922	57.13	368286	163646	5	10	0
30121wG1_15	420	85	5756401	5587425	215	704576	5674311	56.95	292386	133157	7	14	0
30121wG2_37	122	40	5435309	5411276	48	863400	5416597	57.31	372500	190660	5	10	0
30121wG3_49	167	69	5698803	5667545	78	708757	5673017	56.94	214909	169457	10	17	0

30121wH1_16	202	71	5534135	5482261	90	592150	5495341	57.16	183271	115681	10	19	0
30121wH2_40	206	49	5530142	5473689	65	657398	5484626	57.15	294797	195891	6	12	0
30121wH3_50	175	77	5894357	5862119	91	796305	5871236	56.56	214134	95942	10	21	0

Supplementary Table S8. Virulence associated genes in *K. pneumoniae* isolates.

Gene products	Strains			
	KP83	KP124	KP125	KP126
Capsule-related				
capsular synthesis			wzz	
wzi	0	0	0	0
wza	0	0	0	0
wzb	0	0	0	0
wzc	1	1	0	0
wzy	0	0	1	1
wbaP	0	0	0	0
Capsule biosynthesis				
RcsB	1	1	1	1
RmpA	0	0	0	0
RmpA2	0	0	0	0
RmpC	0	0	0	0
RmpD	0	0	0	0
KvrA	0	0	0	0
RfaH	1	1	1	1
KvrB	0	0	0	
Inhibit capsule production				
FNR	1	1	1	1
Fur	1	0	0	1
CRP	1	1	1	1
carbohydrates metabolism				
capsule translocation				
GDP-D-mannose synthesis				
Other sugar-modifying enzymes				
lipopolysaccharide (LPS)				
O-antigen	1	1	1	1
Adhesion	1	1	1	1
Type 3 fimbriae				
fim	1	1	1	1
mrk	0	1	1	1
OMPs		9 OMPs	10	

PalA	1	1	1	1
LppA	1	1	1	1
OmpK35	1	1	1	1
OmpK36	1	1	1	1
KpnO				
T6SS				
PLD1	1	1	1	1
Tle1	1	1	1	0
Iron acquisition				
siderophores including:				
Enterobactin entB	1	1	1	1
yersiniabactin irp2, ybt	0	0	0	0
salmochelin* iroB	0	0	0	0
Aerobactin iucA	1	1	1	0

References

1. Wick, R.R.; Heinz, E.; Holt, K. E.; Wyres, K. L. Kaptive Web: User-Friendly Capsule and Lipopolysaccharide Serotype Prediction for Klebsiella Genomes. *J Clin Microbiol* **2018**, *56* (6). https://doi.org/10.1128/JCM.00197-18.
2. Xu, X.; Li, X.; Luo, M.; Liu, P.; Su, K.; Qing, Y.; Chen, S.; Qiu, J.; Li, Y. Molecular Characterisations of Integrons in Clinical Isolates of Klebsiella Pneumoniae in a Chinese Tertiary Hospital. *Microb Pathog* **2017**, *104*, 164–170. https://doi.org/10.1016/j.micpath.2017.01.035.
